# Micro‐ and Nanomanufacturing for Biomedical Applications and Nanomedicine: A Perspective

**DOI:** 10.1002/smsc.202300039

**Published:** 2023-10-02

**Authors:** Fanjin Wang, Anthony Harker, Mohan Edirisinghe, Maryam Parhizkar

**Affiliations:** ^1^ Department of Mechanical Engineering University College London London WC1E 7JE UK; ^2^ Department of Physics and Astronomy University College London London WC1E 6BT UK; ^3^ School of Pharmacy University College London London WC1N 1AX UK

**Keywords:** healthcare, micromanufacturing, microparticles, nanomanufacturing, nanomedicine, nanoparticles

## Abstract

Almost a century's dedicated research into micro‐ and nanomaterials has yielded fruitful development of preparation methods, achieving fine control over product properties among a broad spectrum of materials. One critical application of these materials lies within the healthcare sector for diagnostic, prophylactic, and therapeutic purposes. However, bench‐to‐bedside translations are still hindered by some unmet demands, especially the scaling‐up from lab‐scale preparation to industry‐level production. The current review recapitulates the strategies of micro‐ and nanomaterial preparation from a holistic viewpoint. The similarities in synthesis and processing methods for various types of materials are highlighted. Furthermore, patents of commercialized nanomedicines are revisited to reveal a solid progress of micro‐ and nanomanufacturing in the last decade. In conclusion, further interdisciplinary research between fields in materials manufacturing is beneficial for the clinical translation and eventually unleashing the power of materials at small dimensions.

## Graphical abstract

The preparation of various types of materials for biomedical applications and nanomedicine is compared. The unique and holistic viewpoint allows comparisons to be made among fields, providing neglected insights. Moreover, manufacturing methods of commercially available nanomedicines are extracted from corresponding patents, presented in an informative tabular format, and discussed to unveil future perspectives of micro‐ and nanomanufacturing.

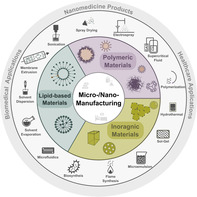

## 1 Introduction

Materials at micrometer or nanometer scale have received tremendous attention in various areas in academic research and industrial application.^[^ ^1^, ^2^ ^]^ Such interest is proved by the ever‐growing revenues in nano‐related industries and the rapidly developing research. Healthcare, as one of the major beneficiaries, is expected to enter a new era of drug delivery, tissue engineering, and diagnostics with nanotechnology.^[^ ^3^, ^4^ ^]^ The term “nanomedicine,” under the definition by European Medicines Agency (EMA), is the application of nanotechnology for medical diagnosis, prophylaxis, or treatment of diseases.^[^ ^5^ ^]^ Nanomedicine is also an area that is growing concomitantly with nanotechnology research. Back in 2005, merely 30 published articles appeared with the topic of nanomedicine.^[^ ^6^ ^]^ The number of publications per year has increased to around 4000 in 2021 according to the Web of Science.^[^ ^7^ ^]^ Decades of research in nanotechnologies are paying off when human beings are confronted with a serious health threat such as SARS‐Cov‐2.^[^ ^3^, ^8^, ^9^ ^]^ The COVID‐19 pandemic in the last 3 years impelled and witnessed the broad application of nanomedicine: the mRNA vaccine by Moderna and BioNTech/Pfizer was delivered by lipid‐based nanoparticles;^[^ ^10^, ^11^ ^]^ and the widely used lateral flow test (LFT) strips were made from surface‐modified gold nanoparticles.^[^ ^12^ ^]^

From the first clinically approved liposome nanoparticle Diprivan in 1989 to the latest two FDA‐approved COVID‐19 vaccines in 2022, around 50 nanomedicine products are approved by Food and Drug Administration (FDA) or European Medicines Agency (EMA) at present.^[^ ^13^ ^]^ It is not difficult to realize that only a limited amount of research has ultimately made the way toward commercialized nanomedicine products in the healthcare industry. Indeed, a series of challenges must be met before it really lives up to the expectation to disruptively change the healthcare industry.^[^ ^14^ ^]^ Among these challenges, a major one is to scale up the production of these materials with micro‐ and nanomanufacturing in compliance with good manufacturing practices (GMP).^[^ ^15^ ^]^ Numerous novel methods of producing micro‐ and nanomaterials have been proposed throughout the years, as evidenced by the constantly updating reviews.^[^ ^16^, ^17^, ^18^ ^]^ However, very few of these methods are translatable into scale‐up industrial settings.^[^ ^18^ ^]^ Even for currently commercialized products like Doxil, manufacturing relies on long and laborious batch processes.

**Figure 1:**
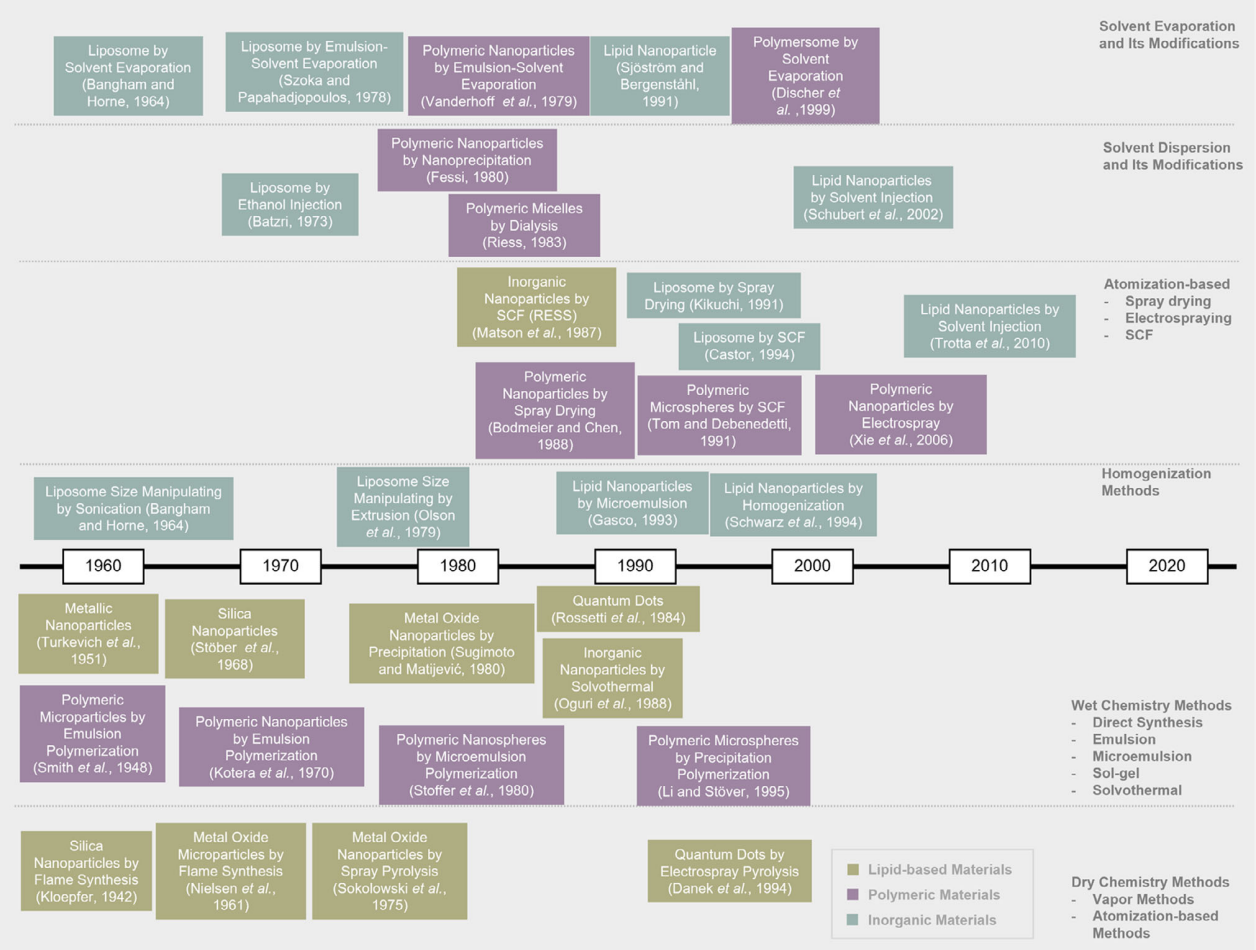
A brief chronology of the development of micro‐ and nanomanufacturing.

Following decades of dedicated research in the field of micro‐ and nanomanufacturing, it is proposed that a retrospective overview would provide valuable insights. In **Figure** **1**, a brief chronology of micro‐ and nanomanufacturing is presented with respect to different materials preparation methods. The timelines are categorized by preparation methods and color‐encoded by the material. This review will focus on methods for small‐batch preparation and scale‐up manufacturing of various micro‐ and nanomaterials used in healthcare. In addition, this review aims to summarize and capture similarities across various research domains in micro‐ and nanomaterials research for healthcare.

### 1.1 Lipid‐Based Materials

**Figure 2:**
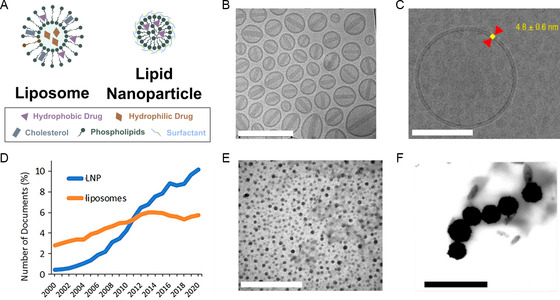
A) A schematic illustration of liposome and LNPs. B) A cryogenic transmission electron microscopy (cryo‐TEM) image of Caelyx, a doxorubicin‐loaded liposome. Scale bar: 200 nm. Reproduced with permission.^[^ ^98^ ^]^ Copyright 2015, Elsevier. C) A close‐up cryo‐TEM image of a drug‐free liposome. Scale bar: 100 nm. Reproduced under the terms of the CC‐BY Creative Commons Attribution 4.0 International license (https://creativecommons.org/licenses/by/4.0).^[^ ^281^ ^]^ Copyright 2021, The Authors, published by MDPI. D) The trend of LNP and liposome‐related documents in CAS Content Collection between 2000 and 2020. Reproduced under the terms of the CC‐BY Creative Commons Attribution 4.0 International license (https://creativecommons.org/licenses/by/4.0).^[^ ^25^ ^]^ Copyright 2021, The Authors, published by American Chemical Society. E) A TEM image of solid lipid nanoparticles loaded with doxorubicin and docosahexaenoic acid. Scale bar: 600 nm. Reproduced with permission.^[^ ^282^ ^]^ Copyright 2012, Elsevier. F) A TEM image of solid lipid nanoparticles loaded with cyclosporin A. Scale bar: 600 nm. Reproduced with permission.

The first few approved nanomedicines, including Doxil (1995) and DaunoXome (1996), are using lipid‐based particles as the drug delivery platform.^[^ ^13^, ^19^, ^20^ ^]^ In fact, hitherto the majority of nanomedicines approved in the clinic are lipid‐based.^[^ ^13^ ^]^ This family of materials has two members: liposomes and lipid nanoparticles (LNPs) (**Figure** **2**A).

#### 1.1.1 Liposomes

Liposome is the most popular drug‐delivery platform in FDA‐approved nanomedicines, according to the study by Anselmo and Mitragotri.^[^ ^13^ ^]^ To date, over 20 commercialized liposome products have emerged on the market, targeting various fields in medicine across oncology to pain management.^[^ ^21^ ^]^ Structurally, liposomes have spherical shapes with one (unilamellar) or multiple (multilamellar) lipid bilayer(s) (Figure 2A). Such characteristic is shown as the dark‐colored edges in TEM images (Figure 2B,C). The size of liposomes is typically at micrometer scale and can be further reduced to 50–200 nm with homogenization techniques.^[^ ^22^ ^]^ The major component of liposomes, the phospholipid, is an amphiphilic molecule with a hydrophilic head and lipophilic tail. Therefore, liposomes are acting as a versatile delivery platform suitable for a wide range of active pharmaceutical ingredients (APIs) with different hydrophilicity. The APIs can find their niche either in hydrophilic environment inside the vesicle or in the lipophilic layer sandwiched in‐between.^[^ ^23^, ^24^ ^]^

#### 1.1.2 LNPs

The second member of lipid‐based nanomedicine is LNPs. The word “lipid nanoparticle” itself was coined after the emergence of nanotechnology, and was around 40 years later than the word “liposome.” Nowadays, the distinction between liposomes and LNPs is somewhat vague. This might be due to similar preparation methods and close major components. In several publications, liposomes were referred to as “the earliest generation of LNPs,” while others strictly define LNPs by the micellar structures in the particle core.^[^ ^25^, ^26^, ^27^ ^]^ As shown by Tenchov et al., the topic about LNPs is receiving an increasing amount of attention in research when compared with liposomes between the years of 2000 and 2020 in the CAS Core Collection (Figure 2D).^[^ ^25^ ^]^ Regardless of the definition, it is widely acknowledged that the development of LNPs began with the attempts to leverage liposomes to deliver nucleic acids.^[^ ^28^ ^]^ The idea of utilizing nuclear acids as therapeutic agents was conceptualized in the 1970s by Friedmann and Roblin.^[^ ^29^ ^]^ However, reaching the site of action of these nucleic acid therapeutics (in the cytoplasm) was never a simple task. The negatively charged phosphate groups presenting in nucleotides can hinder cross‐membrane transport, impeding the activation of these nucleic acid therapeutics in cells.^[^ ^30^ ^]^ Efforts were made to incorporate positively charged lipids to balance out the negative charges from nucleic acids and to optimize the preparation method to best encapsulate and protect these biomolecules against degradation. Solid LNPs, characterized by dark colored particulates shown on TEM images (Figure 2E,F), can be formed with processing technologies such as high‐pressure homogenization or ultrasonication.

### 1.2 Polymeric Materials

Polymeric materials are another large family of materials being used in the healthcare sector. These materials encompass a wide spectrum of natural (e.g., cellulose, cyclodextrin, and silk fibers) and synthetic (e.g., poly (ethylene oxide) [PEO], polystyrene [PS], and polycaprolactone [PCL]) materials.^[^ ^27^, ^31^ ^]^ In a broader sense, polymers were widely used as bulk and composite materials in prosthetics, medical disposable materials, and medical devices in healthcare industry.^[^ ^32^, ^33^, ^34^ ^]^ The application of polymeric materials in COVID‐19 management was reviewed by Singh et al.^[^ ^35^ ^]^ A salient advantage of polymeric materials is the variability. Manipulating primary, secondary, and tertiary structures brings in significant variability in polymers’ characteristics.^[^ ^36^ ^]^ The primary structure is the atomic composition of the polymer. Altering the repeating unit (i.e., the monomer) not only directly impacts mainly the chemical properties like reactivity of the polymer but also varies physical properties like solubility. The secondary structure of a polymer is the size and shape of the long chain. Polymers with different molecular weight behave differently mainly on their physical properties like melting point and solubility. Tertiary structures are interactions between multiple polymer chains. Crystallinity can reflect the tertiary structure of a polymer material and influence the physical properties.^[^ ^36^ ^]^ Thus, polymeric materials can be processed by a range of techniques to satisfy different design and engineering requirements.^[^ ^16^, ^37^ ^]^ The fabrication of polymeric micro‐ and nanomaterials for healthcare can be achieved through a variety of techniques. These techniques can be divided into two categories: those that involve working with preformed polymers, which primarily utilize physical processing methods, and those that leverage chemical processing methods, such as polymerization from monomers. Polymeric materials are mainly processed into the forms of particles and fibers, and are applied for drug delivery, diagnostic, tissue engineering, and wound healing purposes.^[^ ^38^, ^39^, ^40^ ^]^

**Figure 3:**
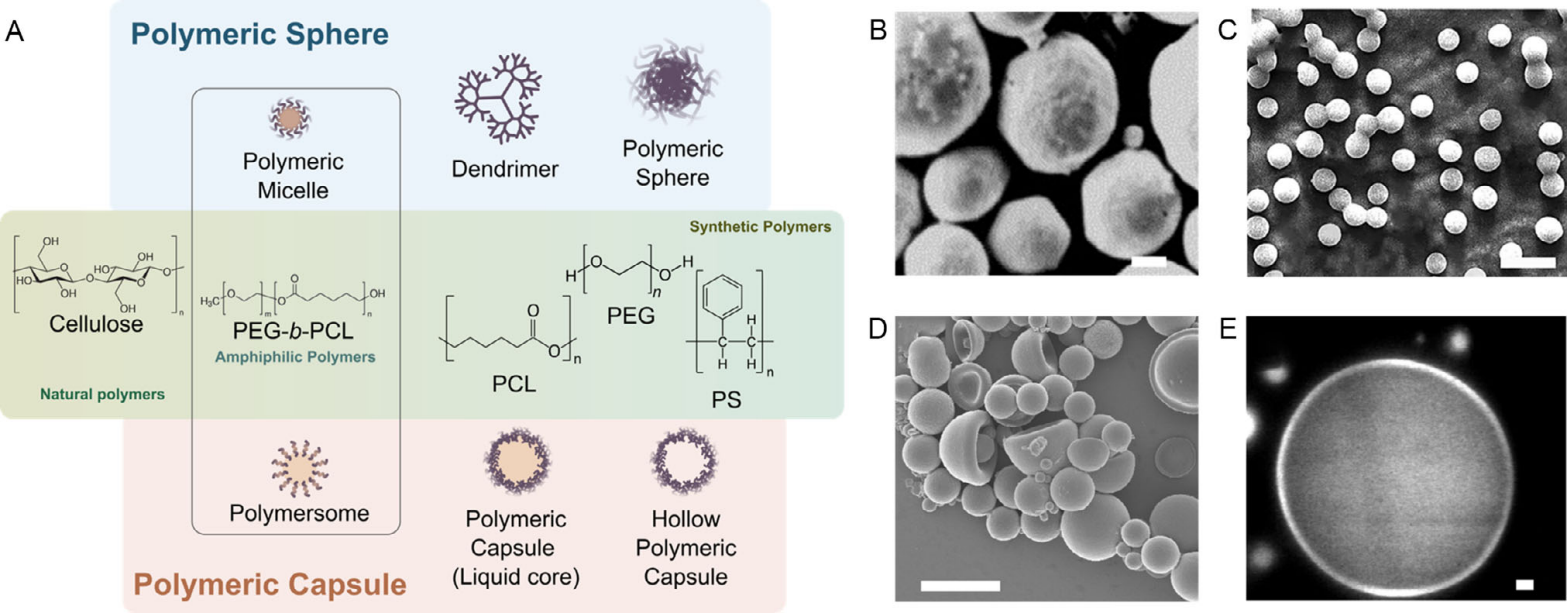
A) A schematic illustration of two kinds of polymeric micro‐ and nanoparticles: polymeric capsule and polymeric sphere. The middle panel shows the chemical structure of polymers selected to prepare these particles. B) A scanning electron microscopy (SEM) image of PLGA microspheres prepared by solvent dispersion. Scale bar: 10 μm. B) Reproduced with permission.^[^ ^283^ ^]^ Copyright 2004, Elsevier. C) An SEM image of PLGA nanospheres synthesized with electrospray. Reproduced with permission.^[^ ^284^ ^]^ Copyright 2021, Elsevier. D) An SEM image of polymeric capsules. The capsules were synthesized from photopolymerization of methacrylate monomers. Reproduced with permission.^[^ ^285^ ^]^ Copyright 2022, American Chemical Society. E) A confocal microscopy image of PEG‐*b*‐PLA polymersomes. Reproduced with permission.^[^ ^54^ ^]^ Copyright 2003, American Chemical Society. Scale bars: 5 μm.

Due to the complexity of polymeric particle structures, it is necessary to rule out some definitions in order to prevent confusion and to thoroughly discuss the preparation methods. Previous studies have demonstrated inconsistencies in the usage of terminologies. In this study, we adopt the classification of polymeric nanoparticles as nanospheres and nanocapsules, as proposed by Mitchell et al.^[^ ^27^ ^]^ Such classification, alongside the common polymers used to prepare micro‐ and nanoparticles, is illustrated in **Figure** **3**A.

#### 1.2.1 Nanospheres

The traditional definition of nanospheres is “particles consisting of solid polymeric matrix.” Polymeric nanospheres exhibit a relatively simple structure and can be formed with a wide range of polymers, including both synthetic and natural polymers. These polymers can be composed of a single type of monomers (homopolymer) or with multiple types of monomers (i.e., copolymer).^[^ ^41^ ^]^ Two SEM images showing the size and morphology of polymeric micro‐ and nanospheres are shown in Figure 3B,C.

##### Dendrimers

Dendrimers form a distinct subclass of nanospheres. They are polymers with a core and many layers of symmetric branches synthesized with special polymerization techniques.^[^ ^42^ ^]^ The molecular weight of dendrimers can vary, while a single dendrimer molecule can be nanosized.^[^ ^42^ ^]^ Such dendrimer nanoparticle has a solid core of polymer matrix. Thus, it is considered as a subcategory of nanospheres in this review. An example of a clinically used dendrimer is SPL7013.^[^ ^43^ ^]^ It is a polylysine‐based dendrimer composed of four layers of lysine branches with strongly anionic disulfonate groups attached to each end. It was approved by the EMA in 2015 for the prevention of vaginal infection as a vaginal gel.^[^ ^44^, ^45^ ^]^

##### Polymeric Micelles

Another subcategory of nanospheres is referred to as polymeric micelles. Polymeric micelles are the aggregation of colloids formed by amphiphilic polymers dissolved above their critical micelle concentration (CMC).^[^ ^46^ ^]^ Typically, these amphiphilic molecules have small molecular weight (<10 000 g mol^−1^) and are block copolymers, comprising a hydrophilic block (e.g., PEO) and a covalently bonded hydrophobic block (e.g., poly(lactic acid) [PLA]) (Figure 3A).^[^ ^47^, ^48^ ^]^ A polymeric micelle can have a size within nanometer scale (<100 nm) and a core self‐assembled from the hydrophobic parts in the polymer chain.^[^ ^41^ ^]^ As reviewed by Houdaihed et al., most clinical trials investigating polymeric micelles are focusing on intravenous delivery of drugs, while the only clinically approved product Cequa is a cyclosporine‐loaded polymeric micelle.^[^ ^49^ ^]^ The nanomicellar technology was patented as NCELL, which contains two amphiphilic polymers (surfactants): PEG‐60 hydrogenated castor oil (HCO‐60) and octoxynol‐40 (OC‐40).^[^ ^50^ ^]^

#### 1.2.2 Nanocapsules

The other family of polymer nanoparticles is nanocapsules. Nanocapsules are vesicles with a polymeric shell and an inner cavity. In some reviews, nanocapsules are strictly referred to those with an oily liquid core, while the vesicles with an aqueous liquid core are called polymersomes.^[^ ^41^ ^]^ Here, we believe it is beneficial to adopt a broader definition for nanocapsules, which does not put constraints on the encapsulated liquid and can also be a hollow cavity.^[^ ^27^, ^31^ ^]^ By adopting such definition, polymeric nanoparticles can be simplified into two categories with similarities and differences (Figure 3A). The building blocks of nanocapsules can be, like nanospheres, from a wide range of polymers if a cavity is created inside (Figure 3D). Pioneering work of fabricating polymer nanocapsules dated back to around 1990 by Fessi et al. where PLA nanocapsules were created.^[^ ^51^ ^]^ The fabrication method, solvent displacement, was further protected with a patent.^[^ ^52^ ^]^ Nowadays, this is recognized as the origin of the so‐called nanoprecipitation method, which is a very important technique to prepare polymer nanoparticles, which will be elaborated in further discussions below.

##### Polymersome

One example of nanocapsules is polymersomes. Polymersomes, take their name from liposomes, are nanocapsules formed with amphiphilic polymers with aqueous liquid entrapped inside.^[^ ^53^ ^]^ Like polymer micelles, in many studies, amphiphilic block copolymers were used to construct the shell (Figure 3A).^[^ ^41^ ^]^ The formation of a micelle or a polymersome structure depends on many factors including the nature of the molecule (especially the ratio of hydrophilic and hydrophobic compartments in copolymers) and the processing technique.^[^ ^41^, ^53^ ^]^ A confocal microscopy photo in Figure 3E showed the distinct bilayer structure through staining with a fluorescent dye.^[^ ^54^ ^]^ Though many polymersomes have been proposed, no known polymersome‐based medicines were clinically investigated.^[^ ^55^, ^56^ ^]^

An important lesson can be learned by ruling out the structural differences of particles: the processing technique plays a critical role in fabricating micro‐ and nanostructures. An example demonstrated by Teixeira et al. highlighted that nanocapsules and nanosphere can be created with the same starting poly (lactic*‐co*‐glycolic acid) (PLGA) polymer but with minor changes in the preparation.^[^ ^57^ ^]^ Another interesting finding is the universal knowledge across different areas in micro‐ and nanomaterials. Taking together the research about lipid‐based and polymeric particles, it is not hard to correlate the comparison between nanocapsules and nanospheres with the distinctions made on liposome and LNPs. Nanocapsules and liposomes both refer to particles with entrapped cavity/solution/liquids whereas nanospheres and LNPs have a relatively solid matrix. In fact, so are the manufacturing methods discussed in the later sections. This highlights the interchangeable knowledge and techniques in materials research.

### 1.3 Inorganic Materials

**Figure 4:**
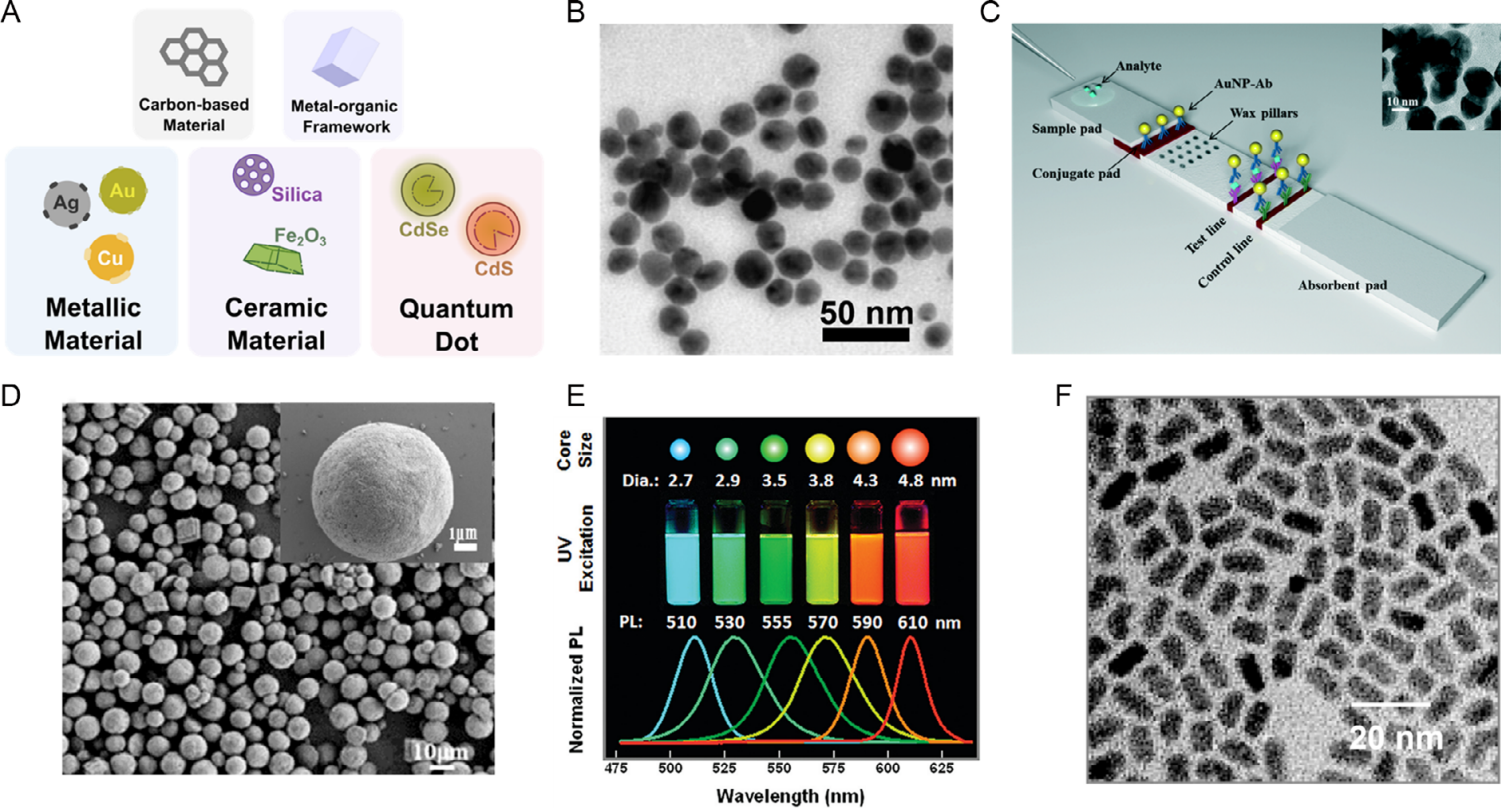
A) A schematic illustration of different classes of inorganic materials used in biomedical applications and nanomedicine. B) A TEM image of silver nanoparticles obtained through biosynthesis. B) Adapted under the terms of the CC‐BY Creative Commons Attribution 4.0 International license (https://creativecommons.org/licenses/by/4.0).^[^ ^286^ ^]^ Copyright 2020, The Authors, published by MDPI. C) An illustration of the lateral flow test strip and its corresponding compartments. (Inset: A TEM image of gold nanoparticles used in the immunoassay). Adapted with permission.^[^ ^77^ ^]^ Copyright, Royal Society of Chemistry. D) A TEM image of porous CaCO_3_ and hydroxyapatite ceramic microspheres. Adapted with permission.^[^ ^287^ ^]^ Copyright 2016, Elsevier. E) Changes in the size of CdSe/ZnS quantum dots are associated with photoluminescent (PL) wavelengths. Reproduced with permission.^[^ ^288^ ^]^ Copyright 2011, American Chemical Society. F) A TEM image of the commercialized CdSe/ZnS quantum dot product: Qdot, Invitrogen. Reproduced under the terms of the CC‐BY Creative Commons Attribution 4.0 International license (https://creativecommons.org/licenses/by/4.0).^[^ ^289^ ^]^ Copyright 2003, The Authors, published by Eaton Publishing Co.

While organic materials gained much research interest in the realm of healthcare applications, a significant body of study investigated the use of inorganic micro‐ and nanomaterials in the healthcare field.^[^ ^27^ ^]^ Inorganic materials have been incorporated in pharmaceutical products as excipients, formulated as health supplements, employed as diagnostic tools, and studied as novel antimicrobial agents.^[^ ^58^, ^59^, ^60^, ^61^, ^62^ ^]^ Compared with other types of materials, inorganic materials have distinct physicochemical properties. To give some examples, heavy metal nanoparticles made from silver or copper exhibit antimicrobial functions.^[^ ^63^, ^64^, ^65^ ^]^ Iron oxide particles, comprising Fe_2_O_3_ or Fe_3_O_4_, possess magnetic properties, which have been leveraged in imaging and diagnostic applications.^[^ ^66^ ^]^ Quantum dots have unique optical and electronic properties due to their special bandgap structures.^[^ ^67^ ^]^ The present study primarily focuses on the synthesis and manufacturing methods of metallic materials, ceramic materials, and quantum dots (**Figure** **4**A). While other materials, such as carbon‐based materials (e.g., graphene, graphene oxides, and carbon nanotubes) and metal–organic frameworks (MOFs), possess unique processing methods, they are not the primary focus of this review. Previous studies have extensively investigated the synthesis, shape‐forming, and manufacturing of these materials, as indicated by a plethora of literature reviews.^[^ ^68^, ^69^, ^70^, ^71^ ^]^ It should be made clear that the current review adopts the strategy to solely refer pure metals and metal alloys as metallic materials, whereas metal compounds are discussed under the category of ceramic materials.^[^ ^59^, ^72^ ^]^ Such classification simplifies the discussion around the preparation methods for corresponding micro‐ and nanomaterials. Typically, metallic micro‐ and nanomaterials are prepared from reduction reactions from solutions. In comparison, metal compounds like metal salts rely on precipitating out metal ions (e.g., using a base) without changing their oxidation state.^[^ ^73^ ^]^

#### 1.3.1 Metallic Materials

Metallic materials, which encompass metals and metal alloys, are a broad category of inorganic materials. Metallic materials have been extensively studied in the field of pharmaceutics, diagnostics, and antimicrobial materials.^[^ ^59^ ^]^ Down to the micro‐ and nanometer scale, the large surface area and the free electrons at the surface of metallic materials brought them unique optical, electronic, and biological properties (Figure 4B).^[^ ^74^, ^75^, ^76^ ^]^ For instance, gold nanoparticles exhibit a red color to the naked eye, as opposed to the characteristic bright yellow color of bulk gold material.^[^ ^74^ ^]^ This phenomenon is a result of the surface plasmon resonance (SPR) effect, which is particularly intense for noble metals. It occurs when electrons at the conduction band of metals interact with incident electromagnetic waves. Such interaction eventually leads to the absorption of light at a specific wavelength.^[^ ^75^ ^]^ Mody et al. reviewed the research in gold and silver nanoparticles and highlighted their applications in in vitro diagnostics.^[^ ^59^ ^]^ In lateral flow strips, gold nanoparticles are bound with antigen‐detecting molecules to produce visual indication of positive or negative result (Figure 4C).^[^ ^77^ ^]^ This sensitive, low‐cost, and user‐friendly lateral flow testing method made indispensable contributions to the management of the COVID‐19 pandemic.^[^ ^78^ ^]^

#### 1.3.2 Ceramic Materials

Ceramic materials for healthcare purposes focus more on three categories: metal oxides, silica‐based materials, and bioceramic materials (e.g., hydroxyapatite and tricalcium phosphate). Iron oxides demonstrate unique magnetic properties that can be leveraged for diagnosis.^[^ ^59^ ^]^ Other metal oxides like titanium oxide are also widely used in personal care as UV absorbents and nontoxic excipients.^[^ ^79^ ^]^ Silica microspheres and nanoparticles are studied as drug delivery and diagnostic platforms due to their highly porous structures.^[^ ^80^, ^81^ ^]^ Some other ceramic micro‐ and nanomaterials are investigated in biomedical implants for their biocompatibility or bioactivity (Figure 4D). Li et al. reviewed various applications of ceramic materials in bone tissue engineering.^[^ ^82^ ^]^ A majority of clinically approved nanomedicines products (via parenteral administration) are supplements to treat iron deficiency rather than being used as a delivery platform. The indications are either for iron replacement therapies or as imaging agents, in which iron is the only approved metal.^[^ ^13^ ^]^ Micro‐ or nanoparticles with other metals like gold and silver are still in the clinical trial stages for efficacy and safety evaluation for use in vivo.^[^ ^13^ ^]^

#### 1.3.3 Quantum Dots

Quantum dots are inorganic materials predominantly comprising of group II to VI or III to V elements. They are semiconductive materials with dimensions of nanometer scale. The reduced size restricts the electrons or holes within the semiconductor and leads to the so‐called quantum confinement effect.^[^ ^83^ ^]^ Such effect brings the material distinctive optical and electronic properties. Among many applications of quantum dots, the efficient PL, characterized by broad excitation and narrow emission spectra, attracts wide research interest as diagnostic agents in healthcare applications (Figure 4E).^[^ ^67^, ^84^ ^]^ Compared with other commonly used organic dyes and fluorescent proteins in biomedical imaging applications, quantum dots have been observed with a tenfold higher brightness and much stronger stability in harsh biological environments.^[^ ^85^, ^86^ ^]^ In addition, the low toxicity profile of quantum dots made them favorable for biomedical applications.^[^ ^87^ ^]^ Previous studies have demonstrated successful imaging with biomarker‐conjugated quantum dots in vitro and in vivo.^[^ ^83^, ^88^ ^]^ For example, CdSe quantum dots have an emission wavelength within visible light range and can be modulated based on the size of the crystal.^[^ ^83^ ^]^ Biomolecules like antibodies, peptides, and DNAs have been conjugated to the surface of various quantum dots for imaging purposes.^[^ ^89^, ^90^, ^91^ ^]^ Furthermore, pH‐sensitive quantum dots have been synthesized as nanosensors to detect the microenvironment.^[^ ^92^ ^]^ Commercialized quantum dot products like Qdot (by Quantum Dot Corp., now acquired by Invitrogen) are also available for research purposes as probes for biomolecules (Figure 4F).^[^ ^93^ ^]^

**Figure 5:**
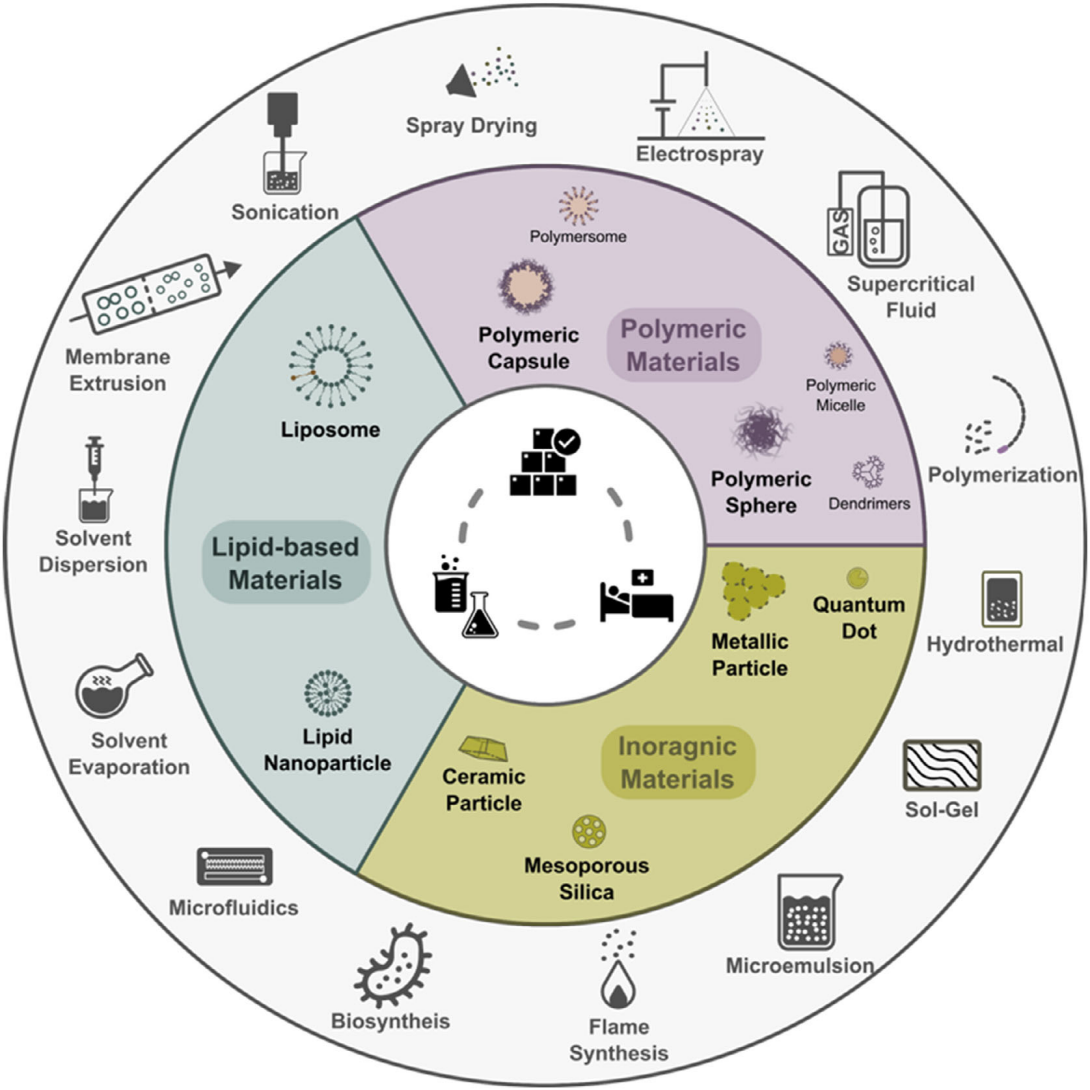
An illustration of micro‐ and nanomaterials and the preparation methods thereof discussed in the current review.

An illustration of different categories of materials and processing methods mentioned in the current review is presented in **Figure** **5**.

## 2 Methods Involving Physical Changes

### 2.1 Solvent Evaporation and Its Modifications

#### 2.1.1 Conventional Solvent Evaporation (Bangham Method and Thin Film Hydration)

**Figure 6:**
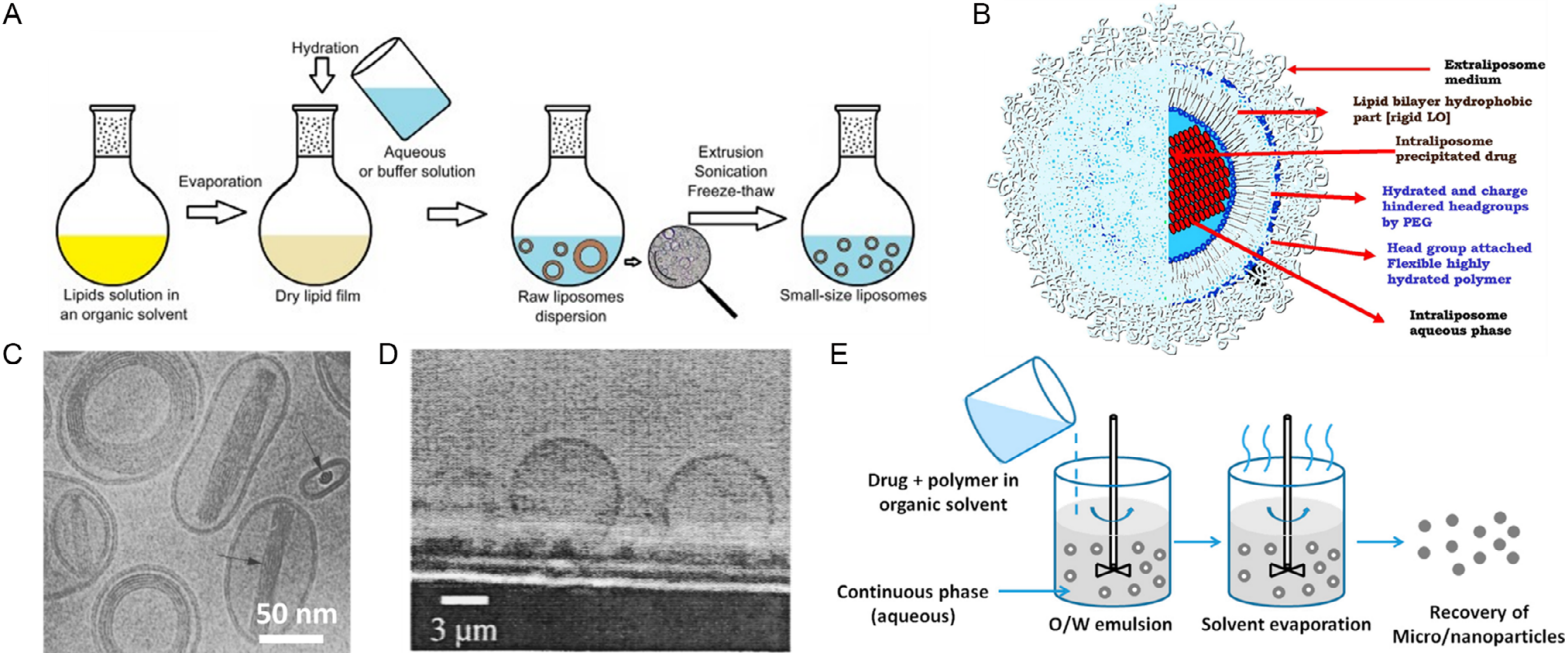
A) An illustration of solvent evaporation method to prepare liposome. Adapted with permission.^[^ ^290^ ^]^ Copyright 2022, Elsevier. B) A cartoon of Doxil based on characterization results. Adapted with permission.^[^ ^19^ ^]^ Copyright 2012, Elsevier. C) A cryo‐TEM image of Myocet, a liposomal formulation of doxorubicin. The thickness of the liposome membrane is approximately 5 nm. Arrows: Doxorubicin crystals. Adapted with permission.^[^ ^100^ ^]^ Copyright 2001, Elsevier. D) A microscopy image of PEO–poly(ethyl ethylene) (PEE) polymersomes prepared by film rehydration method. Adapted with permission.^[^ ^47^ ^]^ Copyright 2001, John Wiley and Sons. E) An illustration of emulsion–solvent evaporation method to prepare polymeric micro‐ and nanoparticles. Adapted under the terms of the CC‐BY Creative Commons Attribution 4.0 International license (https://creativecommons.org/licenses/by/4.0).^[^ ^291^ ^]^ Copyright 2016, The Authors, published by MDPI.

The earliest documented method for the preparation of liposomes, known as the solvent evaporation method, was first discussed in the 1960s by Bangham and Horne.^[^ ^94^ ^]^ This method, which now is referred to as the Bangham method, consists of three key steps: 1) dissolution of lipids in a solvent, 2) removal of the solvent to for a thin lipid film, and 3) rehydration of the film with aqueous buffer solution under agitation. In their initial report, Bangham and Horne utilized chloroform as the solvent for the preparation of liposomes. This choice of solvent is still widely utilized in laboratory settings due to its effectiveness in dissolving lipids and its ease of removal through evaporation.^[^ ^94^ ^]^ A schematic illustration of this method is shown in **Figure** **6**A. Further improvements on this method mainly focused on the agitation or sizing down of liposomes after the rehydration step, which will be discussed in later sections. Other efforts were also made on improving the drug loading efficiency for drugs with variable physicochemical properties, which were reviewed extensively by Shah et al.^[^ ^18^ ^]^

Many pioneers of commercialized liposome products benefited from the solvent evaporation methods in their manufacturing process. Doxil (or Caelyx), approved by FDA in 1995 for the treatment of ovarian cancer and HIV‐associated Kaposi's sarcoma, is a liposomal doxorubicin product originally developed by Sequus Pharmaceuticals, Inc. (which was acquired by ALZA Corporation in 1999, and eventually became part of Johnson & Johnson in 2001) (Figure 6B).^[^ ^95^ ^]^ Patents of Doxil (US4898735A, US5013556A) revealed the application of solvent evaporation method in the manufacturing processes by highlighting the step of rehydrating lipid thin film.^[^ ^96^, ^97^ ^]^ A cryo‐TEM image of Caelyx liposomes has been shown in Figure 2B. It can be seen from the micrograph that after downsizing and homogenization methods, the diameter of these pharmaceutical liposomes was around 100 nm.^[^ ^98^ ^]^ Other examples include DaunoXome (Galen Ltd.), which was approved by FDA in 1996 to treat HIV‐associated Kaposi's sarcoma^[^ ^99^ ^]^ and Myocet (Teva Pharmaceuticals), which was approved by EMA in 2000 to treat breast cancer (Figure 6C).^[^ ^100^ ^]^ More examples of commercialized lipid‐based materials in healthcare can be found in **Table** **1**.

**Table 1:** Preparation methods documented in the patents of currently commercialized nanomedicines

Producta)	Company	API	Main componentsb)	Properties	Delivery technology	Preparation method	Approval [year]	Patent number[s]
Lipid‐based								
Doxil/Caelyx	Janssen	Doxorubicin	PEG, HSPC, cholesterol	Mean size: 100 nm	Liposome	Solvent evaporation	FDA (1995)	US4898735A, US5013556A
DaunoXome	Galen	Doxorubicin	DSPC, cholesterol	Mean size: 45 nm	Liposome	Solvent evaporation	FDA (1996)	US5441745A
Myocet	Teva	Doxorubicin	EPC, cholesterol	Mean size: 150 nm	Liposome	Solvent evaporation – Extrusion	FDA (2000)	US5616341A
Lipodox	Sun Pharma	Doxorubicin	PEG‐DSPE, HSPC, cholesterol	Mean size: <100 nm	Liposome	–	FDA (2013)	–
AmBisome	Gilead	Amphotericin	HSPC, DSPG, cholesterol	Mean size: <100 nm	Liposome	Spray drying	FDA (1990)	US5965156A
DepoCyt	Pacira	Cytarabine	DOPC, DPPG, triolein, cholesterol	Mean size: 10–20 μm	Multivesicular liposome	Emulsification	FDA (1999) – Discontinued	US5723147A US08473019A
Depodur	Pacira	Morphine	DOPC, DPPG, triolein, tricaprylin, cholesterol,	Mean size: 10–20 μm	Multivesicular liposome	Emulsification	FDA (2004) –Discontinued	US5723147A, WO1998033483A1
Exparel	Pacira	Bupivacaine	DEPC, DPPG, tricaprylin, cholesterol	Mean size: 24–31 μm	Multivesicular liposome	Emulsification–solvent evaporation	FDA (2011)	US6132766A, US8182835B2
Nocita	Aratana	Bupivacaine	DEPC, DPPG, tricaprylin, cholesterol	–	Multivesicular liposome	Emulsification–solvent evaporation	FDA (2017)	US8182835B2, US8834921B2, US9205052B2
Marqibo	Acrotech	Vincristine	Sphingomyelin, cholesterol	Mean size: 100 nm	Liposome	Not specified (lyophilized powder)	FDA (2012)	US9801874B2
Arikayce	Insmed	Amykacin	DPPC, cholesterol	Mean size: 300 nm	Liposome	Solvent sispersion (coacervation)	FDA (2018)	US7718189B2, US20080089927A1
Epaxal	Crucell	Hepatitis A virus (inactivated)	Lecithin, cephalin	Mean size: 150 nm	Virosome (liposome with viral spike glycoproteins)	Detergent removal	FDA (2003) – Discontinued	US5565203A, WO199219267
Inflexal V	Crucell	Influenza hemagglutinin protein	Lecithin	Mean size: 150 nm	Virosome	Detergent removal	FDA (1997)	US5879685A
Onivyde	Ipsen	Irinotecan	DSPC, PEG‐DSPE, cholesterol	Mean size: 110 nm	Liposome	Solvent evaporation–extrusion	FDA (2015)	US20180110771A1
Mepact	Takeda	Mifamurtide	POPC, DOPS	Mean size: <10 μm	Liposome	Solvent evaporation	FDA (2004)	US4971802A
Visudyne	Verteporfin	Verteporfin	EPG, DMPC	Mean size: 524 nm^[^ ^297^ ^]^	Liposome	Solvent Evaporation–homogenization (high‐pressure microfluidization)	FDA (2000)	US5707608A
Onpattro	Alnylam	Patisiran	D‐Lin‐MC3‐DMA, PEG‐C‐DMG, DSPC, cholesterol	Mean size: 60–100 nm	Lipid nanoparticle	Solvent dispersion (ethanol injection)–extrusion	FDA (2018)	US8058069B2, US20040142025A1
Vyxeos	Jazz	Daunorubicin and cytarabine	DSPC, DSPG, cholesterol	Mean size: 100 nm	Liposome	Solvent evaporation–extrusion	FDA (2017)	US8431806B2, US10028912B2
Shingrix	GSK	Varicella‐zoster virus (VZV) glycoprotein E	DOPC, cholesterol	Mean size: 100 nm^[^ ^298^ ^]^	Liposome	Solvent evaporation–homogenization (high‐pressure microfluidization)	FDA (2017)	US7939084B1
Mosquirix	GSK	RTS, S antigen	DOPC, MPL, cholesterol	Mean size: 100 nm^[^ ^298^ ^]^	Liposome	Solvent evaporation–homogenization (high‐pressure and high‐shear homogenizer)	FDA (2015)	WO2017102737A1, WO2013041572A1
Comirnaty	BioNTech/Pfizer	mRNA	ALC‐0315, ALC‐0159, DSPC, cholesterol	Mean size: 80–100 nm^[^ ^299^ ^]^	Lipid nanoparticle	Solvent dispersion (ethanol injection); solvent evaporation–extrusion	FDA (2022)	US9950065B2, US10485884B2, US10576146B2
Spikevax	Moderna	mRNA	SM‐102, DSPC, PEG‐DMG, cholesterol	Mean size: 50–200 nm	Lipid nanoparticle	Solvent dispersion (ethanol injection)	FDA (2022)	US10898574B2
SonoVue	Bracco Imaging	Hexafluoride	DSPC, DPPG, PEG4000	Mean size: 2.5 μm	Microbubbles	Sonication	EMA (2001)	US5686060A
Polymeric								
Cequa	Sun Pharma	Cyclosporine	PEG‐60, HCO‐60, OC‐40	Mean size: <30 nm^[^ ^50^ ^]^	Polymeric micelle	Solvent evaporation	FDA (2022)	US9937225B2
Abraxane (albumin particle)	Celgene	Paclitaxel	Human albumin	Mean size: 130 nm	Albumin particle	Emulsion–solvent evaporation	FDA (2005)	US5439686A
Optison (albumin‐stabilized microbubbles)	GE Healthcare	Perflutren microbubbles	Human albumin	Mean size: 2.5–4.5 μm	Albumin particle	Sonication	FDA (1997)	EP0324938B2
Inorganic								
Hensify (hafnium oxide nanoparticle)	Nanobiotix	Hafnium oxide	Hafnium oxide	Mean size: 50 nm	Metal oxide nanoparticle	Direct synthesis (precipitation–calcination)	CE marking (2019)	US10945965B2
CosmoFer (iron dextran colloid)	Pharmacosmos	Iron dextran	Iron hydroxide, dextran	Mean size: <10 nm^[^ ^300^ ^]^	Metal hydroxide colloid	Direct synthesis (neutralization from ferric chloride)	FDA (1992)	US10414831B2
DexFerrum (iron dextran colloid)	American Regent	Iron dextran	Iron hydroxide, dextran	Mean size: 20 nm^[^ ^300^ ^]^	Metal hydroxide colloid	Direct synthesis (neutralization from ferric chloride)	FDA (1996)	US5624668A

a) Abbreviations: HSPC, hydrogenated soy phosphatidylcholine; DSPC, distearoylphosphatidylcholine; EPC, egg phosphatidylcholine; PEG‐DSPE, *N*‐(carbonyl‐methoxypolyethylene glycol 2000)‐1,2‐distearoyl‐*sn*‐glycerol‐3‐phosphoethanolamine; DSPG, distearoylphosphatidylglycerol; DOPC, dioleoylphosphatidylcholine; DPPG, dipalmitoylphosphatidylglycerol; DEPC, dierucoylphosphatidylcholine; POPC, 1‐palmitoyl‐2‐oleoyl‐*sn*‐glycero‐3‐phosphocholine; DOPS, 1,2‐dioleoyl‐*sn*‐glycero‐3‐phospho‐L‐serine; EPG, egg phosphatidylglycerol; DMPC, dimyristoylphosphatidylcholine; DLin‐MC3‐DMA, (6Z,9Z,28Z,31Z)‐heptatriaconta‐6,9,28,31‐tetraen‐19‐yl‐4‐(dimethylamino) butanoate; PEG‐C‐DMG, (α‐(3′‐{[1,2‐di(myristyloxy)propanoxy]carbonylamino}propyl)‐ω‐methoxy, polyoxyethylene); MPL, monophosphoryl lipid A; HCO‐60, hydrogenated castor oil; OC‐40, octoxynol‐40.

b) All information about components and properties of the nanomedicines were obtained from the EMA product information document respectively unless stated otherwise.

Though this method was first discovered and used for producing liposomes, the conventional solvent evaporation approach can also be applied in the preparation of polymeric particles. Synthetic amphiphilic polymers can mimic the structure of phospholipids and self‐assemble into polymeric micelles and polymersomes.^[^ ^53^, ^101^ ^]^ A classical way of preparing polymeric micelles is solvent evaporation.^[^ ^41^, ^102^, ^103^ ^]^ Zhang et al. prepared PLA‐PEG block copolymer micelles by rehydrating the film formed by depleting polymer‐containing acetonitrile with sparging.^[^ ^104^ ^]^ Similarly, Burt et al. followed the same way to prepare poly(lactide*‐co*‐caprolactone) (PLACL)‐PEG copolymer micelles in 1999.^[^ ^105^ ^]^ There are also some reports on preparing these polymersomes with the conventional film rehydration method (Figure 6D).^[^ ^47^, ^106^, ^107^ ^]^ Discher et al. first demonstrated the preparation of polymeresome vesicles in 1999. However, it is recognized that rehydration can be challenging for the film formed by amphiphilic polymers.^[^ ^26^ ^]^ This is partly due to a higher molecular weight and rigidity of synthetic polymers when compared with short‐chain phospholipids.

Further efforts were made to overcome such difficulty by assisting the swelling and detachment of the dried film, including using alternating currents or a precoated hydrogel layer beneath the thin film. Detailed reviews about polymersomes preparation were published by Rideau et al. and Lee et al.^[^ ^26^, ^101^ ^]^ Meanwhile, other scientists observed a way to prepare particles by simply altering steps in the conventional solvent evaporation. This led to the development of the emulsion–solvent evaporation method. It is also called “reverse phase evaporation” in the field of liposome technology, “phase inversion” in polymersome research, and direct “solvent evaporation” in polymeric particle research.^[^ ^101^, ^108^, ^109^ ^]^

#### 2.1.2 Emulsification–Solvent Evaporation

Despite having different names, “emulsification–solvent evaporation” best captures the procedure of this method and indicates that it is a modification of the original solvent evaporation method. Instead of removing the organic solvent to form a thin film and then rehydrating the film, an emulsion is first prepared by mixing the organic solvent (in which polymers or lipids are dissolved) with an aqueous buffer.^[^ ^109^ ^]^ Depending on the property of the solute, sometimes stabilizers (surfactants) are added to help with the emulsification step (Figure 6E).^[^ ^16^, ^108^ ^]^ Emulsification can be achieved by agitation with sonication, vortexing, or simply manual shaking. The level of agitation required in emulsification depends on the material, the desired structure of the particles to be fabricated, and whether there is a postprocessing step of size manipulation. To obtain a suspension of particles in aqueous buffer, the organic solvent is then removed under reduced pressure in a rotary evaporator or under prolonged magnetic stirring with sparging.^[^ ^16^, ^22^ ^]^ This method was first identified as an efficient drug loading method for liposome preparation by Szoka and Papahadjopoulos in 1978.^[^ ^110^ ^]^ Almost at a similar time, this method was reported in the field of polymeric particle preparation and was patented in 1979 by Vanderhoff et al.^[^ ^111^ ^]^ As recorded in the patent, this idea was not inspired from liposome research. In fact, it originated from another important way to prepare polymeric nanoparticles—emulsion polymerization—which will be covered in later sections involving chemical reactions.^[^ ^112^ ^]^ The application of this method in polymeric materials was rejuvenated in the early 2000s by Gurny et al.^[^ ^113^ ^]^ They prepared drug‐loaded particles from a biodegradable polymer PLA and demonstrated its superiority in storage stability, biocompatibility, and prolonged drug release profile. Since then, a plethora of polymeric particles including polymersomes were prepared with preform polymers for various applications in healthcare, especially for drug delivery.^[^ ^101^ ^]^

### 2.2 Solvent Dispersion and Its Modifications

#### 2.2.1 Conventional Solvent Dispersion (Solvent Displacement, Ethanol Injection, Nanoprecipitation)

**Figure 7:**
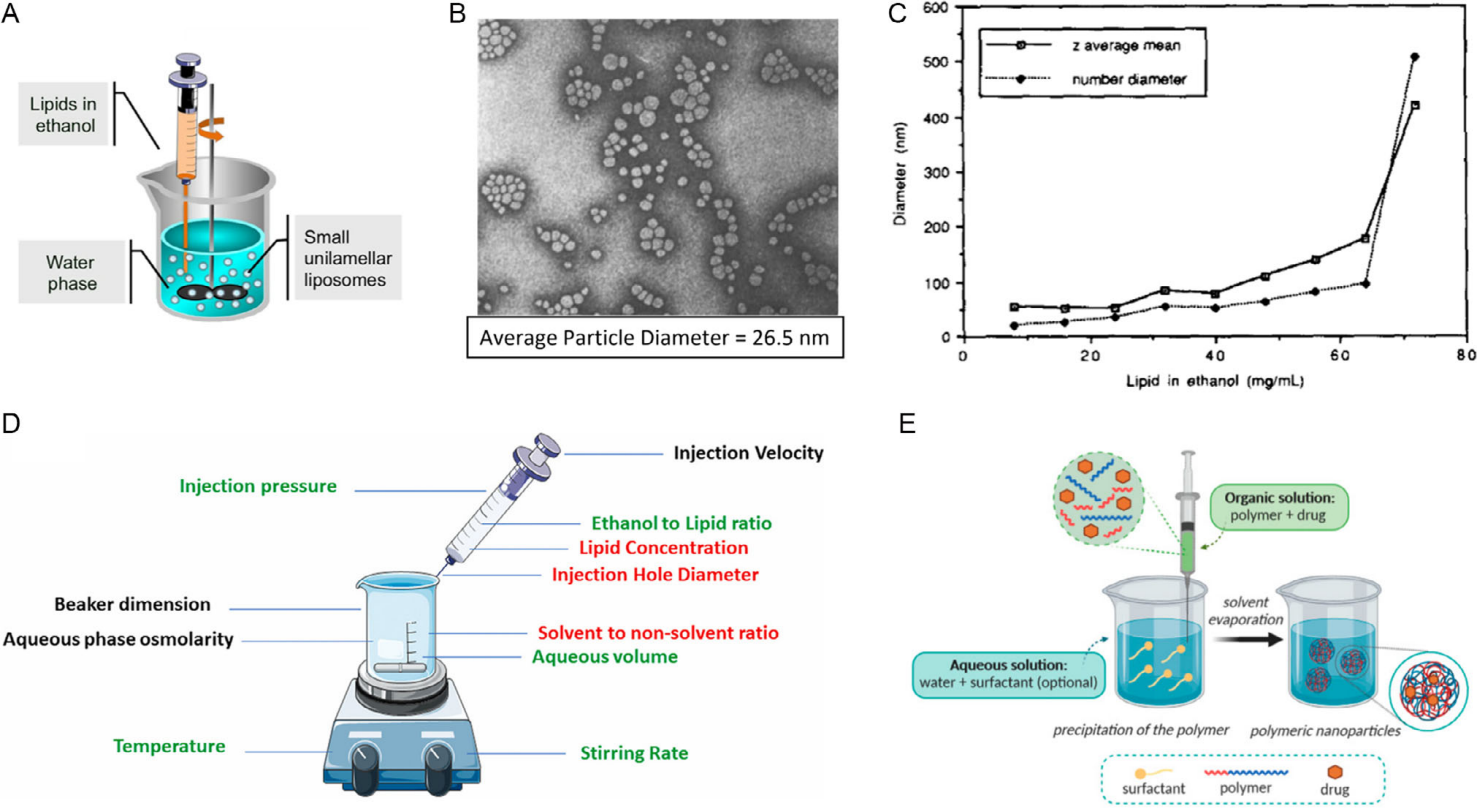
A) An illustration of ethanol injection method to prepare liposome. Adapted under the terms of the CC‐BY Creative Commons Attribution 4.0 International license (https://creativecommons.org/licenses/by/4.0).^[^ ^292^ ^]^ Copyright 2016, The Authors, published by Springer Nature. B) A negative SEM image of the liposomes in Batzri and Korn's original publication. The instrumental magnification was 36 200×. Error bar was not stated in the original publication. The liposomes were determined to have an average diameter of 26.5 nm. Adapted with permission.^[^ ^114^ ^]^ Copyright 1973, Elsevier. C) The diameters of liposomes obtained from injecting different concentrations of lipid–ethanol solutions. Adapted with permission.^[^ ^115^ ^]^ Copyright 1993, Elsevier. D) Parameters that affect the solvent dispersion process. Black colored parameters indicate no effect on the size of the particle. Green refers to positive effect and red shows negative effect. Adapted with permission.^[^ ^117^ ^]^ Copyright 2021, Elsevier. E) An illustration of solvent displacement method to prepare polymeric particles. Adapted under the terms of the CC‐BY Creative Commons Attribution 4.0 International license (https://creativecommons.org/licenses/by/4.0).^[^ ^293^ ^]^ Copyright 2020, The Authors, published by MDPI.

Solvent dispersion was proposed around 10 years ago after the traditional solvent evaporation method for liposome preparation.^[^ ^22^, ^114^ ^]^ The workflow of solvent dispersion can be briefly summarized into the following steps: 1) dissolving polymers or lipids, along with lipophilic components (e.g., hydrophobic drugs), in water‐miscible solvents (typically alcoholic solvents like methanol, ethanol, isopropanol, or a mixture thereof); 2) dissolving hydrophilic components (e.g., excipients, nucleic acids, and hydrophilic drugs) in a water‐based buffer solution; 3) mixing the two compartments under specific conditions (e.g., agitation).^[^ ^115^ ^]^ A specific case of solvent dispersion in fabricating lipid‐based materials, where ethanol was used as the solvent, was called ethanol injection (**Figure** **7**A). The ethanol injection method for liposome fabrication was first put forward by Batzri and Korn in 1973 (Figure 7B).^[^ ^114^ ^]^ Originally, the lipid–ethanol solution (lipid concentration of 36.5 mm) was rapidly injected into sodium chloride buffer with a Hamilton syringe. The liposomes were further purified with ultrafiltration under rapid stirring with nitrogen. The spirit of solvent dispersion utilizes the change in lipid solubility during the mixing of solvent and antisolvent. Later, Kermer et al. revealed that it was not the injection velocity but rather the lipid concentration in ethanol that posed significant influence on the size of liposomes produced with this method.^[^ ^116^ ^]^ Pons et al. conducted further experiments to evaluate the effect of parameters on the size and stability of the liposomes made from ethanol injection method (Figure 7C).^[^ ^115^ ^]^ It turned out that multiple processing parameters can have an impact on the diameter of the particles prepared from this method, as reviewed by Gouda and co‐workers (Figure 7D).^[^ ^117^ ^]^ These findings in solvent dispersion reflected the potential of replacing the batch‐based and energy‐consuming steps in solvent evaporation (e.g., heating and drying the lipid film in vacuo) to a cheaper and continuous manufacturing paradigm. On top of the benefits in manufacturing, ethanol, as a relatively nontoxic class 3 solvent, is easier to deal with when compared with other organic solvents like chloroform and methanol (both class 2 solvents in industrial guidelines) used in thin film preparation.^[^ ^118^, ^119^ ^]^ In fact, both FDA‐approved COVID‐19 vaccines Comirnaty and Spikevax highlighted ethanol injection as their manufacturing method in their patents.^[^ ^120^, ^121^, ^122^, ^123^ ^]^ It can be seen in Table 1 that all four liposome products approved after 2018 endorsed this new technology in their patents.^[^ ^13^ ^]^

Solvent dispersion can also be used to prepare polymeric particles, although the name of the method was changed to solvent displacement.^[^ ^16^ ^]^ Nanoprecipitation, developed in the late 1980s by Fessi et al. to produce polymeric nanoparticles, was originally referred to as the solvent displacement method.^[^ ^51^ ^]^ An illustration of the method is shown in Figure 7 E. In the original publication, 125 mg of PLA was first dissolved in 25 mL of acetone with a model drug, indomethacin. Then, the acetone solution was poured into 50 mL of surfactant‐containing water under moderate stirring. The resulting mixture, as described in the article, immediately turned milky opaque. The suspension was identified to be nanocapsules of PLA around 229 nm. When compared with solvent dispersion (ethanol injection) developed in 1970s, it is clear that solvent displacement, or the nanoprecipitation method, shares the same underlying principle of particle formation. Indeed, the experimental setup of both methods requires simple components such as a syringe, a magnetic stirrer, and a beaker.^[^ ^31^ ^]^ The method became a very popular fabrication route for polymeric nanocapsules at lab scale due to its simplicity.

#### 2.2.2 Modified Solvent Dispersion

As in the case of solvent evaporation, research continues to explore and improve the solvent dispersion method. It is worth noting that a few other strategies that developed later still followed the principle of solvent dispersion. Here, these methods are introduced as variations of solvent dispersion. To elaborate further, the principles utilized in solvent dispersion were observed and studied in detail.^[^ ^124^, ^125^ ^]^ In 2005, Ganachaud and Katz named it after the Ouzo effect, which originally described the formation of an emulsion when water was added to ouzo liquor (a flavored alcohol containing a hydrophobic essential oil).^[^ ^124^ ^]^ The Ouzo effect precisely captured the ethanol injection (solvent dispersion) process when the lipid–ethanol solution was injected into water. As the lipid molecule is amphiphilic, the “emulsion” would be constituted by the lipophilic part of lipids or by water‐encapsulating liposomes. The case is similar in the preparation of polymeric materials. Therefore, a broader definition of solvent dispersion can be described as methods to mix solvents which eventually impair the solubility of the solute and result in the formation of particles, due to the change in polarity (ethanol injection, ether injection, and nanoprecipitation), ionic strength (salting‐out and ionotropic gelation), or other physicochemical properties.^[^ ^126^, ^127^ ^]^

#### 2.2.3 Dialysis Method (Osmosis‐Based Method)

A slightly different method is called the direct dialysis method. Like solvent dispersion, the solute (e.g., polymers) is first dissolved in organic solvents and kept in a dialysis bag (membrane), followed by submerging into aqueous solutions under stirring.^[^ ^128^ ^]^ By doing so, the organic solvent, which is miscible with water, gradually diffuses across the dialysis membrane and the solute molecules precipitate due to the change of solubility. Several previous researchers agreed to list direct dialysis method as a variation under nanoprecipitation.^[^ ^17^, ^127^, ^129^ ^]^ In fact, this method has long been utilized in the preparation of polymeric micelles back in 1990s, as reviewed by Riess.^[^ ^130^ ^]^The dialysis method has also been used to prepare polymeric particles like PLGA and polymethylmethacrylate (PMMA) nanospheres.^[^ ^131^ ^]^

### 2.3 Atomization‐Based Methods

All previously mentioned methods prepare lipid and polymeric particles as a suspension in liquid. As an improvement, there are a few methods that have been proposed to directly fabricate particles in one step via atomizing the material‐containing liquids and obtaining dried particulates in situ due to the evaporation of solvents.^[^ ^132^ ^]^ In this sense, the atomization‐based methods are extensions of the solvent evaporation method. These atomization‐based methods were used either in combination with the previously mentioned methods or independently as a simple fabrication step.

#### 2.3.1 Spray Drying

**Figure 8:**
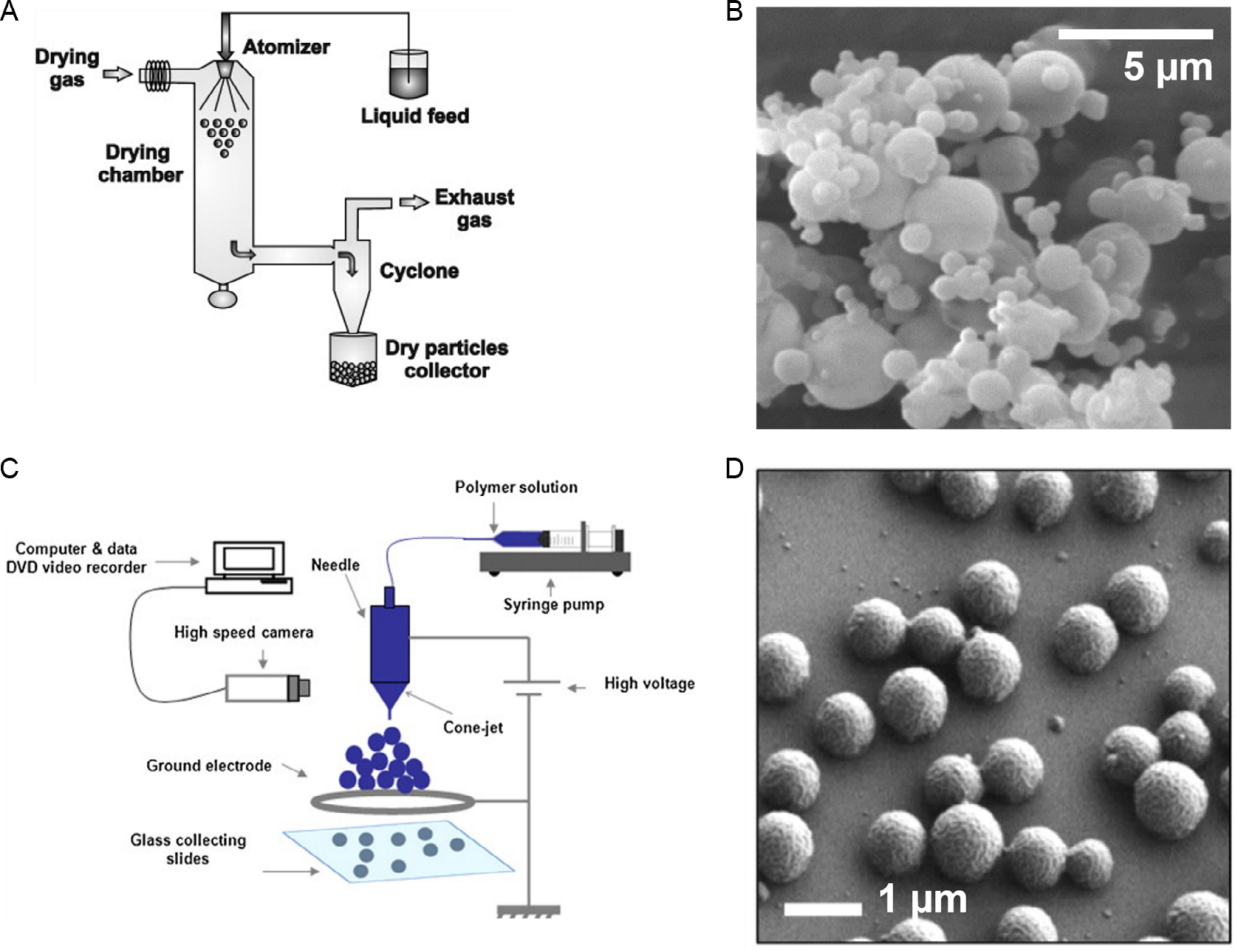
A) A diagram of the process and equipment for a spray drying device. Adapted with permission.^[^ ^294^ ^]^ Copyright 2015, Elsevier. B) An SEM of PLGA particles obtained by spray drying. Adapted under the terms of the CC‐BY Creative Commons Attribution 4.0 International license (https://creativecommons.org/licenses/by/4.0).^[^ ^140^ ^]^ Copyright 2021, The Authors, published by MDPI. C) A setup of electrospraying device. Adapted with permission.^[^ ^295^ ^]^ Copyright 2011, Elsevier. D) PLGA microparticles obtained by electrospraying. Adapted with permission.^[^ ^146^ ^]^ Copyright 2014, Elsevier.

Spray drying is a well‐documented method to prepare pharmaceutical ingredients into particles.^[^ ^133^ ^]^ Briefly, the solute components are dissolved in a volatile solvent like chloroform or dichloromethane. Then, the organic solution is pumped into an atomization head. With the help of hot inlet compressed air, the liquid is sprayed from the atomization head, the solvent is evaporated, and the solute components are dried and collected (**Figure** **8**A).

The spray drying method to prepare liposomes was reported back in 1991 by Kikuchi et al.^[^ ^134^ ^]^ Liposomes were formed by reconstructing these powder products. This method was further studied by Skalko‐Basnet et al. to prepare drug‐loaded liposomes.^[^ ^135^ ^]^ However, it was observed that the powder obtained through spray dry was only a preliminary form of liposome and were essentially large solid lipid particles at submillimeter scale (≈500 μm).^[^ ^136^ ^]^ After reconstitution with aqueous buffer under continuous stirring for 45 min at 400 rpm, liposomes of 200–500 nm diameter were observed.^[^ ^135^, ^136^ ^]^

Similarly, spray drying was used for the preparation of polymeric particles. Spray drying of polymers, alongside APIs or food ingredients, was extensively studied in the 1990s. The area was reviewed in detail by Vehring.^[^ ^133^ ^]^ Polymers, like ethyl cellulose, PLA, and PLGA, were prepared as microspheres in many encapsulation applications (Figure 8B).^[^ ^137^, ^138^, ^139^, ^140^ ^]^ In the past, particles were mainly at microscale in the applications. This is because the size of the particles made by spray drying is directly related to the size of the droplets formed and thus closely linked to the spray drying device. Recent improvements in spray drying technology enabled the spraying of submicron droplets with specifically designed vibrating mesh spray orifices; this allowed the preparation of submicron particles through spray drying.^[^ ^141^, ^142^ ^]^

Inorganic nanoparticles can also be fabricated with spray drying. However, most applications are related to chemical preparation and the heat decomposition of precursors, which will be discussed in a separate section.

#### 2.3.2 Electrospray (Electrohydrodynamic Atomization)

Electrospray, also called Electrohydrodynamic atomization (EHDA), refers to the technique that atomizes liquid droplets with the help of an electric field.^[^ ^143^ ^]^ It is similar to spray drying in the fact that the formation of particles happens in situ with the evaporation of solvents in the atomized droplets. Unlike spray drying which relies on pressurized airflow, electrospraying atomizes liquid under the electric field. The droplets are charged, distorted, and disintegrated in the air due to the repulsive electrostatic forces.^[^ ^144^ ^]^ Electrospray was first used for the analysis of biological samples in mass spectrometry in the late 1990s.^[^ ^145^ ^]^ Later, the usage of electrospray extended to the preparation of micro‐ and nanostructures.^[^ ^143^ ^]^ It was observed as a versatile technique that forms much finer droplets with narrowly dispersed size when compared with spray drying.^[^ ^132^ ^]^ In addition, electrospraying has a much simpler setup (Figure 8C). Thus, it is regarded as a promising technique for the manufacturing of particles in the next decades that could be favored from an industrial production perspective.^[^ ^132^ ^]^

Electrospray technology is relatively new, and its applications have primarily focused on the preparation of polymeric particles, rather than lipid‐based materials.^[^ ^143^ ^]^ Extensive attempts have been made to electrospray a wide range of synthetic and natural polymers including PLGA, PCL, and chitosan (Figure 8D).^[^ ^132^, ^146^, ^147^ ^]^ The versatility of electrospray allows manipulation of product properties through altering the processing parameters. This leads to the change of polymeric particles size distributions, ranging from 100 nm to 10 μm.^[^ ^146^ ^]^

Only a few reports investigated processing lipid‐based materials with electrospraying. Studies by Bussano et al. and Trotta et al. demonstrated the fabrication of LNPs through electrospray, with the successful incorporation of insulin into the particles.^[^ ^148^, ^149^ ^]^ Additionally, Eltayeb et al. described the preparation of solid lipid nanoparticles with similar formulation, with a reduction in particle size below 100 nm.^[^ ^150^ ^]^ Furthermore, Duong et al. reported the one‐step preparation of liposomes with a coaxial electrospray device.^[^ ^151^ ^]^

#### 2.3.3 Supercritical Fluid Method

**Figure 9:**
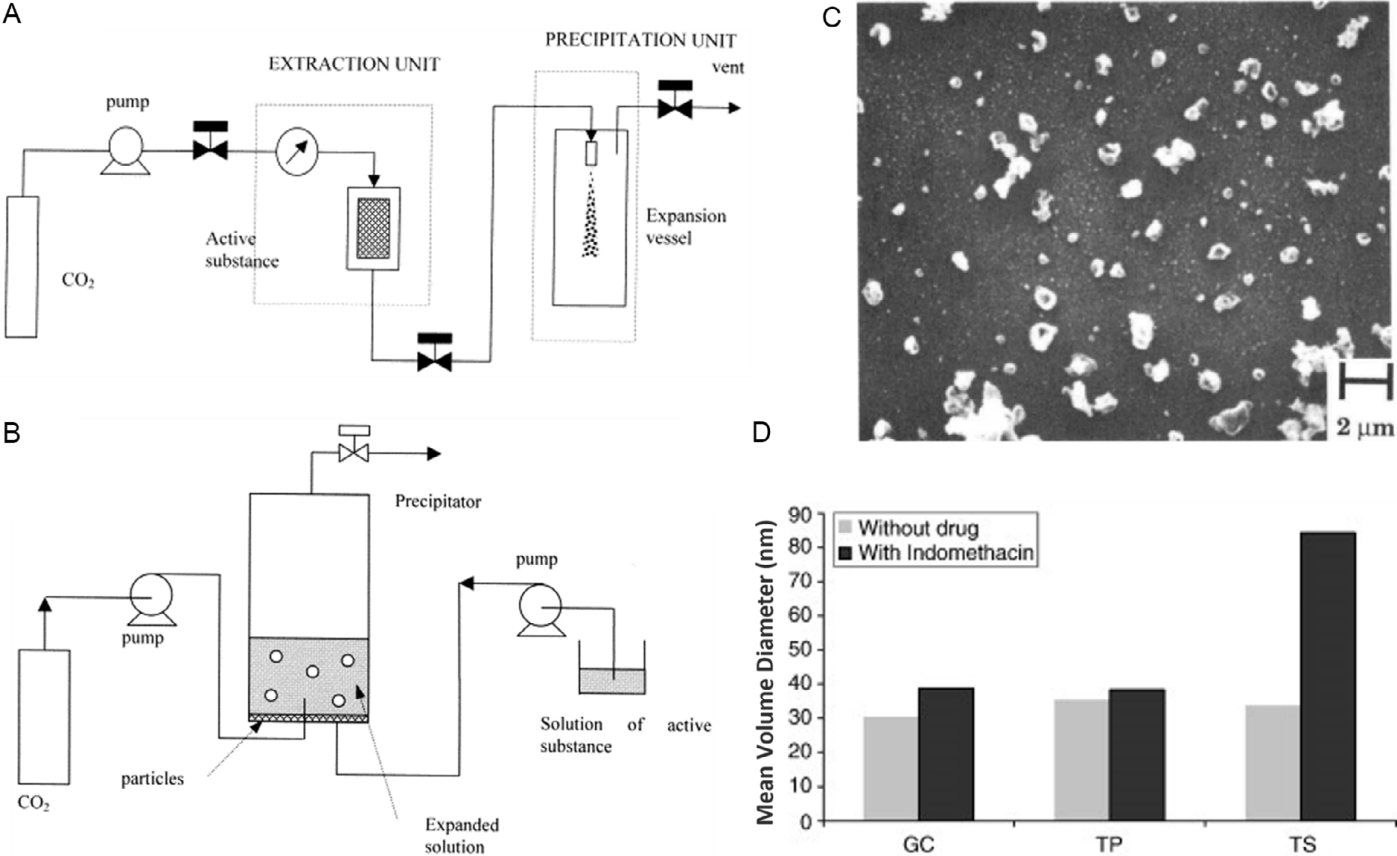
A,B) Schematic illustrations of RESS (A) and SAS (B) equipment that leverage supercritical fluids to produce micro‐ and nanomaterials. A,B) Reproduced with permission.^[^ ^165^ ^]^ Copyright 2001, Elsevier. C) An SEM image of PLA nanoparticle produced with RESS process. Reproduced with permission.^[^ ^157^ ^]^ Copyright 1994, American Chemical Society. D) Mean diameter of different solid lipid nanoparticles produced with SAS method (GC, Gelucire 50/13; TP, Tripalmitin; TS, Tristearin). Adapted with permission.^[^ ^164^ ^]^ Copyright 2007, Elsevier.

Yet another atomization‐based method is the supercritical fluid method. The study of supercritical fluids to dissolve solids dates back to 1879 by Hannay and Hogarth.^[^ ^152^ ^]^ Multiple preparation methods of particles with supercritical fluid have been proposed and can be classified based on the role of the supercritical fluid.^[^ ^153^, ^154^ ^]^ It is suggested that the simplest method is the rapid expansion of supercritical solutions (RESS), proposed by Matson et al. in 1987.^[^ ^155^ ^]^ RESS represents a family of methods where the supercritical fluid is used as the solvent of the material being processed.^[^ ^153^ ^]^ In RESS, the supercritical fluid solution is depressurized into a collector in a controlled manner, generating small particles of the material (**Figure** **9**A).^[^ ^156^ ^]^ The size of the particles was typically at micrometer scale and sometimes were at submicrometer scale (Figure 9B).^[^ ^156^, ^157^ ^]^ Other methods were also developed for materials that were less soluble in supercritical fluids. In these methods, the supercritical fluid was treated as an antisolvent of the material (e.g., supercritical antisolvent [SAS] method) (Figure 9C). It was used in combination with the previously mentioned methods like solvent dispersion to induce the precipitation of the solute.^[^ ^153^ ^]^ The supercritical fluid method is believed to allow continuous processing at an industrial level if designed properly.^[^ ^158^ ^]^ Also, in some applications, it can avoid the use of organic solvents, making it favorable for healthcare applications. A salient disadvantage of this method is the demanding price: the expensive operational cost and complicated high‐pressure apparatus required. In addition, it is difficult to gain control over the size and size distribution of the particles fabricated through supercritical fluid methods.^[^ ^154^, ^159^ ^]^

Meure et al. reviewed the developments in using supercritical fluid for the preparation of liposomes.^[^ ^160^ ^]^ In 1990s, both groups of Castor in the United States and Frederiksen et al. in Switzerland proposed improved methods based on RESS to prepare liposomes.^[^ ^161^, ^162^ ^]^ In their publications, nanometer scale liposomes were obtained through depressurizing the lipid–ethanol–supercritical CO_2_ solution into an aqueous medium. Direct RESS preparations of lipid microparticles were recorded by both Castor and Badens.^[^ ^161^, ^163^ ^]^ Chattopadhyay et al. demonstrated the production of LNPs around 30 nm with an improved SAS method (Figure 9D).^[^ ^164^ ^]^ The manufacturing of polymeric particles with supercritical fluids was also extensively studied.^[^ ^165^ ^]^ Yeo et al. reviewed the area of polymeric particle formation with supercritical fluid methods.^[^ ^166^ ^]^ As the solubility of most macromolecules in supercritical CO_2_ was poor, cosolvents were added to help with the RESS process.^[^ ^166^, ^167^ ^]^ In the 1990s, microparticle fabrication using supercritical fluids was investigated with a plethora of polymers like PEG, PLA, PLGA, and PMMA.^[^ ^168^, ^169^, ^170^ ^]^ More recently, polymeric nanoparticles were fabricated with modified methods.^[^ ^171^ ^]^

### 2.4 Size Manipulation Methods for Lipid‐Based Materials

**Figure 10:**
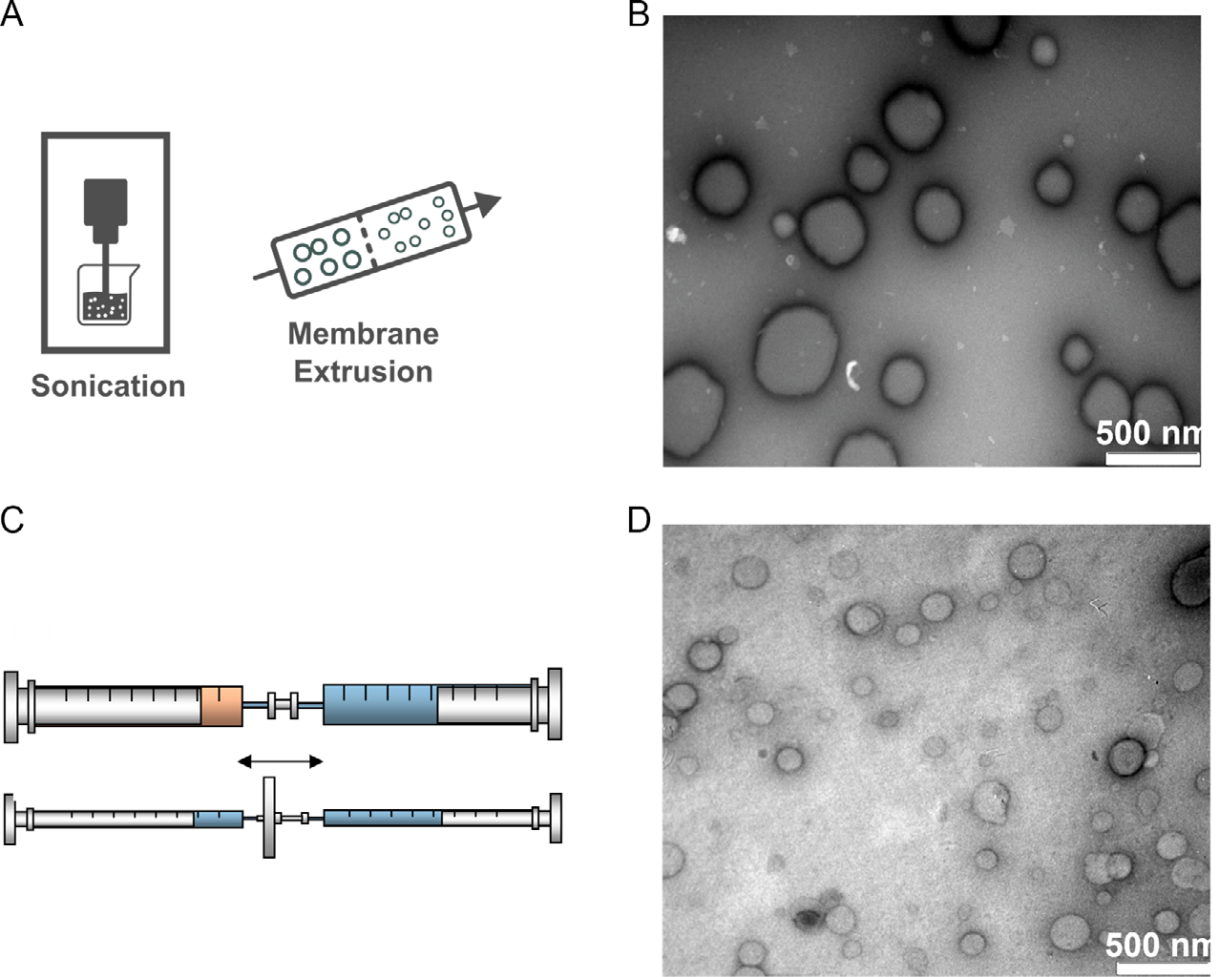
A) Schematic illustrations of the sonication and membrane extrusion methods. B) Liposomes produced with solvent evaporation followed by 20 min bath sonication. B) Adapted with permission.^[^ ^175^ ^]^ Copyright 2007, American Chemical Society. C) A simple setup composed of two syringes and a membrane filter for membrane extrusion method to prepare lipid nanoemulsions. Top: Premixing the lipid and surfactant solution. Bottom: Extrusion by pushing the liquid between two syringes back and forth. Adapted with permission.^[^ ^178^ ^]^ Copyright 2016, Elsevier. D) Liposomes produced with membrane extrusion with solvent evaporation followed by extrusion from filter. Adapted with permission.^[^ ^175^ ^]^ Copyright 2007, American Chemical Society.

Liposomes and LNPs formed with the aforementioned methods are often not in a desired size or size distribution. Thus, further size reduction and homogenization methods were applied in the preparation of these particles. For drug delivery purposes, the size and size distribution have critical impact on the biodistribution of the particle and furthermore the pharmacokinetics and pharmacodynamics of the encapsulated drug.^[^ ^172^ ^]^ Ideally, the size of the particle should be above 20 nm to avoid renal clearance and be controlled within a certain range to achieve optimized retaining in blood stream.^[^ ^173^ ^]^ For liposomes and LNPs, nearly all commercialized products are below 200 nm unless tailored to the target (e.g., Mepact‐targeting macrophages) or not administered through intravenous infusion (DepoCyt—intraventricular injection, DepoDur—epidural injection, and Exparel—local injection).^[^ ^21^ ^]^ There are two popular methods used for size manipulation: 1) sonication and 2) extrusion (**Figure** **10**A). Typically, these methods are applied to lipid‐based materials but can also process certain polymeric materials (like polymeric micelles and polymersomes).^[^ ^174^ ^]^

#### 2.4.1 Sonication

Sonication is the oldest method for liposomes’ size manipulation. It was first documented alongside Bangham's report of solvent evaporation method in 1960s where an 80 W sonicator was used to treat the 1 mL suspension of liposomes.^[^ ^94^ ^]^ Devices for sonication can be bath‐based or probe‐based. Figure 10B showed an example of liposomes prepared from solvent evaporation followed by a 20 min bath sonication.^[^ ^175^ ^]^ Sonication baths deliver less uniform and weaker energy when compared with sonication probes. Probe sonication is a widely reported method to produce stable and uniform liposomes in laboratories.^[^ ^18^ ^]^ A drawback of the sonication method include the potential degradation of the lipids or the loaded drugs due to the heat and vibration and the contamination by metals from the sonication probe.^[^ ^176^ ^]^

#### 2.4.2 Extrusion

As shown in Table 1, extrusion is the most popular method currently used in the liposome industry. This method was first reported by Olson et al. in 1979.^[^ ^177^ ^]^ Extruding the suspension of liposomes prepared through solvent evaporation or solvent dispersion through a membrane (e.g., polycarbonate membrane) could easily manipulate the liposomes’ size and size distribution (Figure 1C).^[^ ^178^ ^]^ Depending on the desired size for liposomes, membrane with a specific pore size could be selected.^[^ ^179^ ^]^ As shown in Figure 10B,D, the authors managed to prepare liposomes with similar drug delivery properties from sonication and membrane extrusion.^[^ ^175^ ^]^ Both liposomes yielded similar drug release properties albeit having different diameters (extruded: 112 nm, sonicated: 28 nm). Compared with other methods, extrusion is a preferred method because it is relatively energy‐saving and induces less lipid oxidation.^[^ ^26^, ^180^ ^]^ However, the suspension needs to be heated above the transition temperature of lipid components during the extrusion process. Otherwise, the poor mobility of the lipid molecules can clog the polycarbonate membrane and lead to ruptures of the membrane.^[^ ^180^ ^]^

## 3 Methods Involving Chemical Changes

Previous sections captured the mainstream and novel fabrication methods of particles at micro‐ and nanometer size. In this part of the review, preparation methods that involve chemical changes of the materials to form particles are discussed. These methods are also referred to as synthetic methods because they include chemical synthesis in the workflow instead of working with preformed materials. Among three kinds of micro‐ and nanomaterials discussed previously, lipid‐based materials are seldom prepared through chemical synthesis. Typically, the components of lipid‐based materials like lipids and cholesterols are synthesized before assembling into particles. By contrast, polymeric and inorganic particles are formed through chemical synthesis, especially for the inorganic particles.^[^ ^73^, ^181^ ^]^ Here, depending on the synthetic process, the methods are categorized into wet chemistry methods and dry (vapor) chemistry methods.

### 3.1 Wet Chemistry Methods

Wet chemistry methods involve a series of reactions that take place in solvents. The preparation of micro‐ and nanoparticles can start in a homogeneous or a heterogeneous solution. In a homogeneous solution, the formation of solid particles by adding other reagents could directly lead to precipitation or the formation of colloidal suspension. These methods are referred to as direct synthesis methods in this review. Various methods including (co)precipitation, hydrothermal, solvothermal, and sol–gel methods fall into this category. Moreover, chemical reactions that take place in heterogeneous solutions like emulsions can also lead to particle formation in the discrete phase. These emulsion‐based methods are discussed separately because they are more universal methods regardless of the material being fabricated whereas direct synthesis methods are designed for specific inorganic/polymeric materials.^[^ ^182^ ^]^ An additional step of drying is required to obtain a powder form of particles from wet chemistry methods.

#### 3.1.1 Direct Synthesis

**Figure 11:**
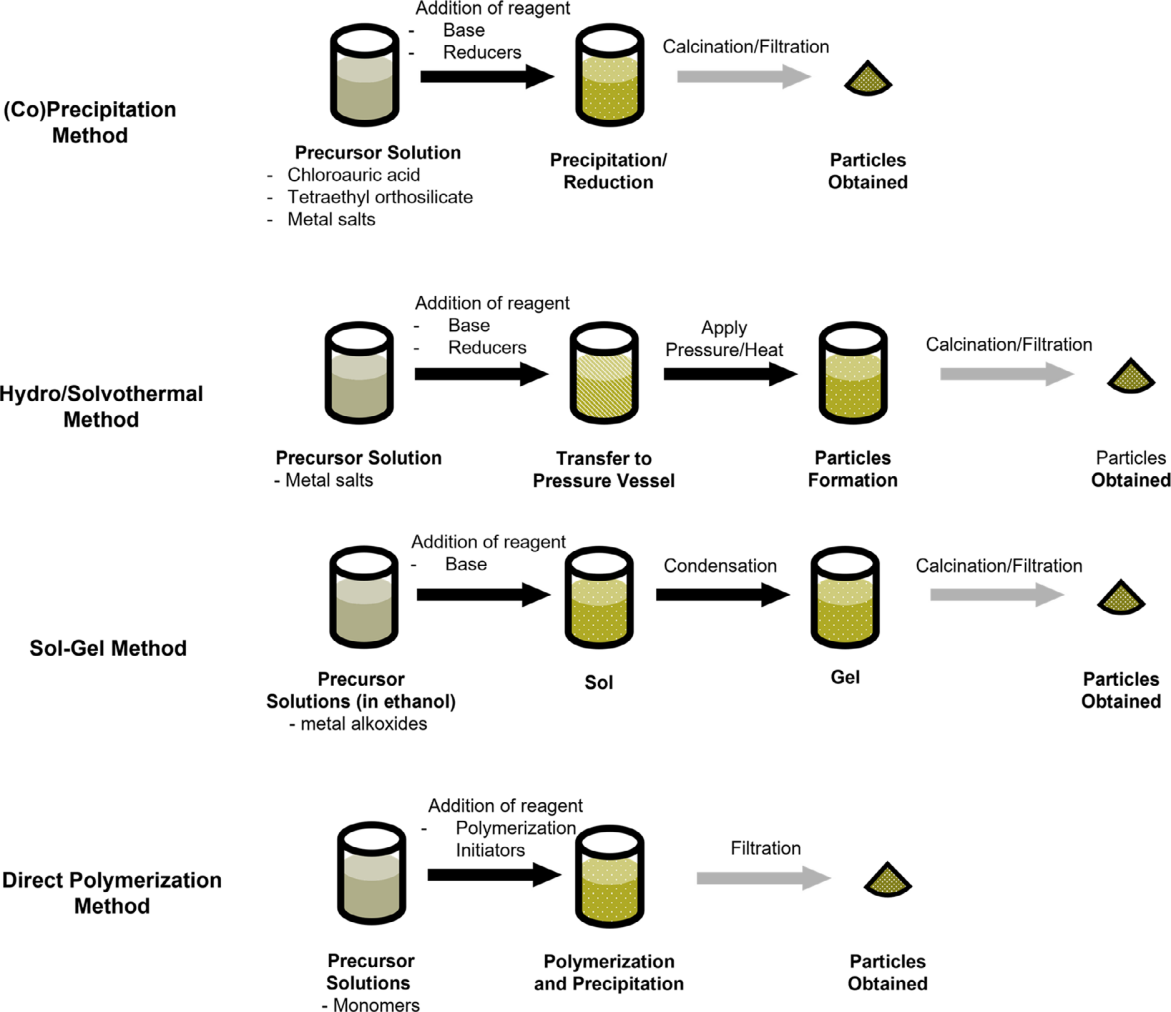
A schematic illustration of the procedures and reagents in direct synthesis methods of micro‐ and nanomaterials.

The simplest way to synthesize micro‐ or nanoparticles is the direct synthesis method. This method encompasses a wide range of reactions and methods that start in a homogenized liquid phase (**Figure** **11**). Depending on the material being processed, different reactions like polymerization, reduction, or hydrolysis are chosen. With the addition of reagents, solubilized precursors turn into insoluble products and small particles are formed via nucleation, growth, and aggregation mechanisms.^[^ ^183^ ^]^ Sometimes, surfactants or stabilizers are added to help stabilize the particles formed and avoid further aggregation/agglomeration. These methods are further classified into precipitation, sol–gel, and solvothermal methods.

##### Direct Precipitation/Precipitation–Calcination Method

**Figure 12:**
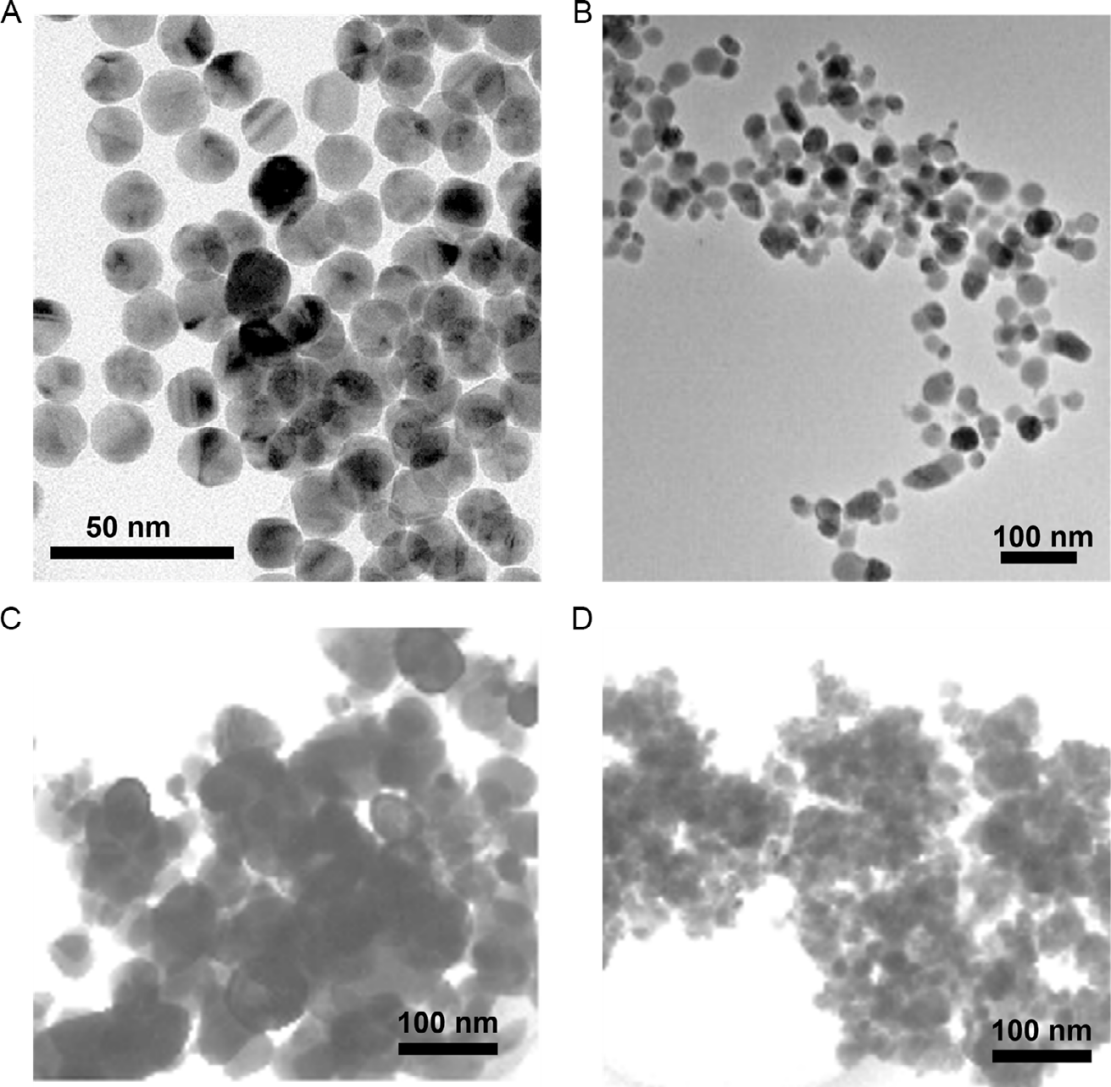
TEM images of nanoparticles prepared by direct precipitation from their salt solutions. A) Gold nanoparticles prepared with Turkevich method. Adapted under the terms of the CC‐BY Creative Commons Attribution 4.0 International license (https://creativecommons.org/licenses/by/4.0).^[^ ^296^ ^]^ Copyright 2020, The Authors, published by Hosokawa Powder Technology Foundation. B) Silver nanoparticles obtained from reducing silver nitrate with aniline. Adapted with permission.^[^ ^186^ ^]^ Copyright 2010, Elsevier. C) Zinc oxide and D) tin oxide nanoparticles precipitated from metal chlorides with ammonium hydroxide. C,D) Adapted with permission.^[^ ^187^ ^]^ Copyright 2002, Elsevier.

The precipitation of metal nanoparticles can be achieved by reducing them from their salt form with appropriate reducers (Figure 11). The famous Turkevich method to prepare gold nanoparticles is a paradigm for the precipitation method.^[^ ^184^ ^]^ Originally, sodium citrate was used as the reducing agent (and as the stabilizer) to reduce the boiling chloroauric acid (HAuCl_4_) solution to gold nanoparticles, yielding nanoparticles around 20 nm (**Figure** **12**A). Similarly, silver nanoparticles can be prepared through adding sodium borohydrides to silver nitrate (AgNO_3_) solution Figure 12B.^[^ ^185^, ^186^ ^]^ Extensive work has been done in this field that explored various materials including metals and metal alloys, reducing agents like borohydrides and sugars, solvents like water, and organic solvents.^[^ ^73^ ^]^

Fabrication of metal oxide nanoparticles is relatively more sophisticated. It can be achieved by calcinating precursors like metal hydroxide or carbonates obtained through precipitation from adding a base into a metal salt solution or through the hydrolysis of metal alkoxides. The precipitated metal hydroxides are further calcinated at a relatively low temperature (≈400 °C) so that decomposition happens to the precipitated hydroxides while the sintering of the material is minimized to avoid agglomeration of particles.^[^ ^73^ ^]^ A wide range of metal oxide nanoparticles has been prepared in this way including titanium dioxide, zinc dioxide, and ceramics that contain multiple metal ions.^[^ ^187^ ^]^

##### Hydrothermal/Solvothermal Method

A very similar variant to this precipitation–calcination method is the famous hydrothermal or solvothermal method, where the hydrothermal method uses water as the solvent and the solvothermal method uses organic solvents (Figure 11).^[^ ^188^ ^]^ In both methods, the steps include: 1) dissolve a soluble salt of the metal in the solvent, 2) adjust the pH by adding base, 3) transfer all components into a pressure vessel, and 4) keep in an autoclave at a temperature higher than the boiling point of the solvent.^[^ ^189^, ^190^ ^]^ Byrappa and Adschiri reviewed the applications of hydrothermal and solvothermal methods for nanotechnology.^[^ ^189^ ^]^ They noted that the research in hydrothermal and solvothermal methods was first restricted by characterization techniques and was mainly focused on the preparation of bulk crystals instead of fine particles of materials. It was not until the 1990s that the research about the preparation of micro‐ and nanomaterials with hydrothermal and solvothermal methods emerged.^[^ ^191^ ^]^ Currently, hydrothermal and solvothermal methods are popular methods to prepare inorganic materials, including quartz, metal oxides, and ceramic materials, with finely controlled size and morphology.

##### Sol–Gel Method

**Figure 13:**
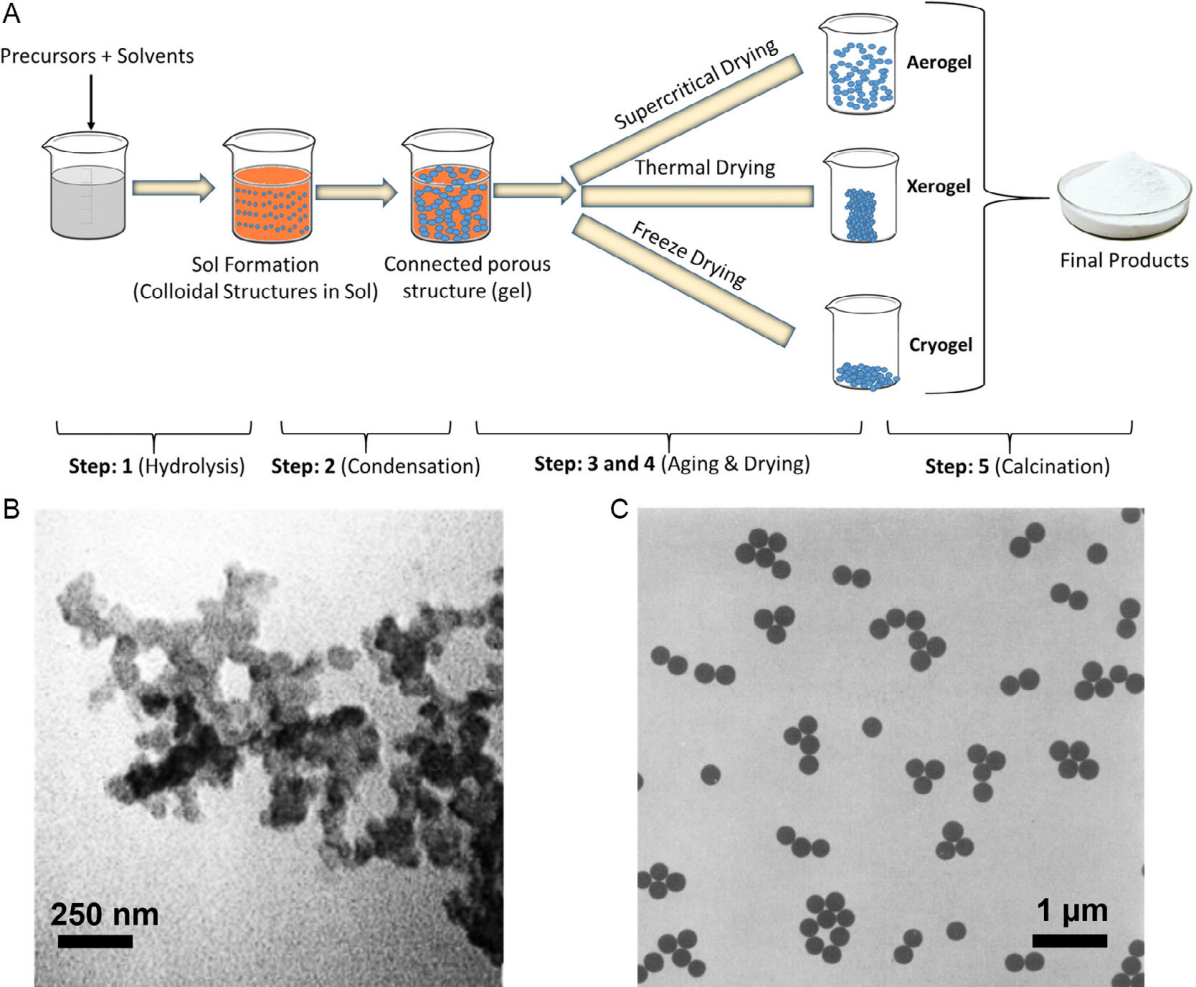
A) An illustration of the steps in the sol–gel method. Reproduced with permission.^[^ ^194^ ^]^ Copyright 2020, Springer Nature. B) A TEM image of iron oxide nanoparticles obtained from hydrolysis of metalorganic precursors. Adapted with permission.^[^ ^195^ ^]^ Copyright 2001, Springer Nature. C) A TEM image of silica microparticles prepared by the Stöber method. Adapted with permission.^[^ ^196^ ^]^ Copyright 1968, Elsevier.

Another similar method is the sol–gel method.^[^ ^192^ ^]^ In this method, metal alkoxides are first dissolved in an alcohol and are then converted into metal hydroxides through hydrolyzing with water (Figure 11). This leads to the formation of a colloidal suspension. In some reports, direct filtration was used to harvest amorphous nanoparticles from the ‘sol’.^[^ ^193^, ^194^ ^]^ In other reports, further conversion of the ‘sol’ into the ‘gel’ was achieved with continuous stirring while condensation happened at adjacent molecules. To prepare nanoparticle powders, the dried gel is calcinated, like the treatment in the precipitation–calcination method (**Figure** **13**A **)**.^[^ ^73^, ^193^ ^]^ For example, nanoparticles of iron oxides including γ‐Fe_2_O_3_ and Fe_3_O_4_ were prepared by Gun'ko et al. from the hydrolysis of iron metalorganic precursors. The particles were filtered and further calcinated at 300 °C (Figure 13B).^[^ ^195^ ^]^ Direct filtration without further calcination may produce amorphous nanoparticles whereas drying and calcination lead to more ordered or crystalized nanoparticles.^[^ ^73^ ^]^ Other than metal alkoxides, silica nanoparticles can also be prepared via such route. In fact, this is one of the earliest sol–gel methods and also known as the Stöber method. Monodispersed silica nanoparticles can be prepared through the hydrolysis of silicon alkoxide (specifically, tetraethyl orthosilicate [TEOS]) (Figure 12C).^[^ ^196^ ^]^Notably, using sol–gel to produce nanoparticles is merely one of the many applications of this method. Before the drying step, the gel can also be coated onto other surfaces to prepare dense ceramic films. Brinker and Scherer discussed a wide range of applications of the sol–gel method, including for the preparation of inorganic fibers, composites, coatings, and membranes.^[^ ^193^ ^]^

##### Precipitation Polymerization (Direct Precipitation of Polymers)

Direct synthesis methods of polymeric micro‐ and nanoparticles that only require the precursor monomer, the solvent, and the initiator in a one‐phase solvent system are less commonly used than the two‐phase emulsion‐based methods.^[^ ^197^, ^198^ ^]^ This is partly due to the nature of polymerization. If the monomer is fully dissolved in a solvent or the bulk monomer is directly used for polymerization, the polymerization will not be terminated until the depletion of monomers, so the product will likely to be bulk material rather than micro‐ or nanoparticles. However, an exception here is precipitation polymerization. In 1995, Li and Stöver proposed the so‐called precipitation polymerization where poly(divinylbenzene) microspheres were obtained by directly initiating divinylbenzene polymerization in acetonitrile.^[^ ^199^ ^]^ Without adding any surfactants, the dissolved monomer in acetonitrile gradually turned into insoluble polymer and precipitated, yielding microspheres around 2–5 μm. Since then, precipitation polymerization was further investigated by other researchers and became an important method to produce molecularly imprinted polymer (MIP) microparticles.^[^ ^200^, ^201^ ^]^ Li et al. summarized different variations of precipitation polymerization to fabricate polymeric particles.^[^ ^202^ ^]^

#### 3.1.2 Emulsion and Microemulsion Method

The microemulsion method, or more broadly the emulsion method, is the most versatile chemical synthesis method used throughout different disciplines of materials science. Both polymeric and inorganic micro‐ and nanomaterials can be fabricated through this method combined with chemical reactions.^[^ ^203^ ^]^ Fundamentally, microemulsion is a two‐phase system with surfactants supporting the interface, bringing it to a low‐viscous, transparent, and stable system.^[^ ^204^ ^]^ The discrete phase, served as microreactors at micrometer scale, is generated through appropriate agitation methods like ultrasound.^[^ ^204^ ^]^ The study of microemulsion was started by Schulman et al. in the 1950s. And the term “microemulsion” was coined in their 1959 article.^[^ ^205^ ^]^ Today, this thriving field developed many variants including water‐in‐oil, oil‐in‐water, and supercritical microemulsions. In fact, the microemulsion method can also be utilized to generate particles from preformed materials, as demonstrated in different studies.^[^ ^206^, ^207^ ^]^ However, it is more popular as a versatile platform to synthesize various nanoparticles, combined with chemical reactions selected with reaction mechanisms and precursors. Metals like gold, silver, copper, and platinum nanoparticles have been synthesized in microemulsions with chemical reductions.^[^ ^208^ ^]^ Micro‐ and nanoparticles of metal oxides have been prepared by hydrolysis in water‐in‐oil microemulsions.^[^ ^209^, ^210^ ^]^ Studies were also conducted on the preparation of silica particles with microemulsions.^[^ ^211^, ^212^ ^]^ Quantum dots and semiconductor particles like ZnS, ZnSe, and CdSe were reported to be produced using this technique.^[^ ^213^, ^214^, ^215^ ^]^ Attempts were also made to synthesize nanosized ceramics like hydroxyapatite nanoparticles with the microemulsion method.^[^ ^216^ ^]^

When it comes to organic polymeric materials, there is a long history of using emulsion polymerization to prepare polymer particles, which was commercialized in 1930s to prepare polymer latex.^[^ ^217^ ^]^ However, this traditional method typically produces particles with a large size distribution from micrometers to a few nanometers.^[^ ^217^ ^]^ In contrast, the microemulsion method allowed fine control over the particle size distribution.

### 3.2 Dry (Gas) Chemistry Methods

Unlike wet chemical methods that require an additional step to dry the fabricated particles, dry (gas) chemical methods directly obtain particles in the gas phase.^[^ ^218^ ^]^ This can be achieved by either gas‐to‐particle conversion or particle‐to‐particle conversion.^[^ ^219^ ^]^ Gas‐to‐particle conversion is achieved by using precursors in vapor form and chemical reactions directly convert them into smoke/aerosol. Particle‐to‐particle conversion leverages atomization methods to nebulize small liquid droplets via spraying or electrospraying. However, these gas‐based methods require specific equipment that can handle high pressure and high temperatures. Thus, it is less practical at a laboratory level but could be favored in large‐scale industrial applications because no further separation steps are needed and the continuous nature of these manufacturing routes makes them more attractive in an industrial setting.^[^ ^218^ ^]^ Also, these methods are mostly designed for inorganic particles that are less temperature sensitive.

#### 3.2.1 Vapor‐Based Methods (Chemical Vapor Synthesis, Flame Synthesis, and Plasma Synthesis)

Vapor‐based methods, as the name suggests, utilize the gas form of precursors to fabricate particles. This method is very popular in the synthesis of metal oxide micro‐ and nanoparticles where metal–organic compounds are chosen as precursors.^[^ ^218^, ^220^ ^]^ Metal–organic compounds like metal alkoxides (e.g., titanium tetraisopropoxide [TTIP]) or metal acetates (e.g., zinc acetate) will go through heat decomposition or combustion in furnace, flame, plasma, or laser.^[^ ^219^, ^221^ ^]^ The large market (multiple billions of dollars per year) of combusted “fume” metal oxides like silica, titania, and alumina was created and products are widely used in the industry as catalysts, reinforcement agents, and pigments.^[^ ^222^ ^]^ More recently, industrially synthesized micro‐ and nanoparticles through vapor‐based methods are utilized in healthcare scenarios as filler materials in dental and bone tissue engineering applications and also was further functionalized for drug delivery and diagnostic purposes.^[^ ^219^ ^]^

#### 3.2.2 Atomization‐Based Methods (Spray pyrolysis, Electrospray pyrolysis, and Flame spray pyrolysis)

Atomization‐based methods utilize the capability of spraying devices to produce micro‐ or nanosized droplets that contain precursors of particles.^[^ ^223^ ^]^ These liquid particles can go through further heating or combustion to yield dry powders below micrometer scale.^[^ ^224^ ^]^ As reviewed by Teoh et al., metal, metal oxides, other ceramic materials, and composite materials have been successfully synthesized with this method.^[^ ^225^ ^]^ However, the difficulty to spray narrowly distributed droplets led to the unsatisfactory polydispersity of particles fabricated with flame spray pyrolysis.^[^ ^223^, ^225^ ^]^ As previously mentioned, the incorporation of electrospray avoided the coagulation problem and could produce fine droplets.^[^ ^226^ ^]^ Electrospray pyrolysis was proved to generate much finer and narrowly distributed metal, metal oxide, and other inorganic nanoparticles.^[^ ^227^, ^228^, ^229^ ^]^

## 4 Other Manufacturing Methods

### 4.1 Microfluidics

**Figure 14:**
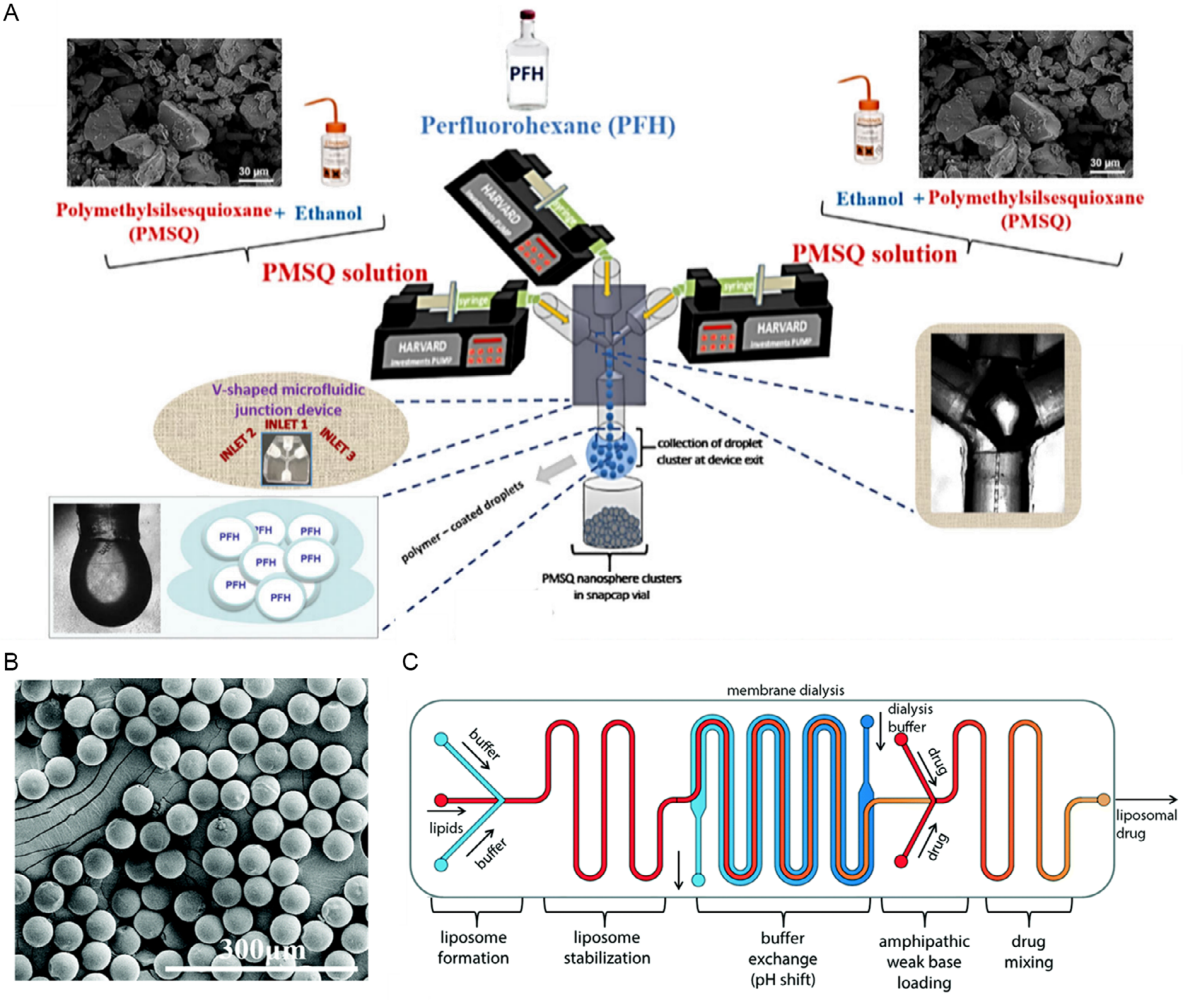
A) An illustration of the method to prepare PMSQ nanospheres with microfluidics Adapted under the terms of the CC‐BY Creative Commons Attribution 4.0 International license (https://creativecommons.org/licenses/by/4.0).^[^ ^237^ ^]^ Copyright 2014 The Authors, published by Springer Nature. B) An SEM image of Janus microparticles made from PCL/PLGA using microfluidics. Adapted with permission.^[^ ^238^ ^]^ Copyright 2015, Royal Society of Chemistry. C) A microfluidics lab‐on‐a‐chip design to prepare liposomes. Adapted with permission.^[^ ^241^ ^]^ Copyright 2014, Royal Society of Chemistry.

Microfluidics are essentially systems designed to manipulate minute amounts of liquids with the help of micrometer‐scale channels on chips.^[^ ^230^ ^]^ Since its development in the early 1960s, microfluidics systems have revolutionized many areas in chemical synthesis and analysis.^[^ ^231^, ^232^ ^]^ Such popularity comes from an innate merit of microfluidics—being able to process a minute amount of sample. A wide variety of channel layouts have been designed and implemented to execute operations that were previously done in round bottom flasks like transporting, mixing, and separation. In addition, temperature manipulation, electronic devices, and detection units can be easily integrated into the microfluidic system, which allows for a unified synthesis and characterization workflow in a controlled environment.^[^ ^230^, ^231^ ^]^ More recently, advanced computational and modeling methods like machine learning and computational fluid dynamics (CFD) accelerated the microfluids research.^[^ ^233^, ^234^ ^]^ Liu et al. reviewed microfluidics with the emphasis of drug development applications.^[^ ^235^ ^]^ They highlighted the potential use of microfluidic chips for lab‐on‐a‐chip diagnosis and for the synthesis of drug delivery agents. Li et al. summarized the application of microfluidics for microparticles generation where the implemented methods on microfluidic chips were modified from original flask‐based synthesis methods.^[^ ^236^ ^]^ Indeed, the versatility made microfluidics a powerful tool that can be integrated into, if not completely replace, the manufacturing process of materials. To give some examples, the direct synthesis of inorganic materials like metal oxides through direct precipitation method relies on the mixing of metal precursors and a base solution.^[^ ^73^ ^]^ The ethanol injection methods to prepare LNPs also requires the mixing of ethanol solution of lipids with aqueous buffer.^[^ ^115^ ^]^ Kucuk and Edirisinghe demonstrated the preparation of polymethylsilsesquioxane (PMSQ) nanospheres with microfluidics channels (**Figure** **14**A).^[^ ^237^ ^]^ The PMSQ nanospheres, with diameters around 80–920 nm, were formed on the surface of a volatile liquid perfluorohexane. Li et al. utilized microfluidics system to produce Janus polymeric microparticles with an average diameter of 28 μm by the simple mixing of polymer solutions with an aqueous antisolvent (Figure 14B).^[^ ^238^ ^]^ These simple mixing processes can be done in a precisely controlled and continuous manner. Furthermore, another saliant advantage of these microfluidics systems made it a promising technology for not only laboratory research but also mass production—parallelization. Parallelization of these well‐controlled systems offers an alternative to simply scaling up the reactor in mass production.^[^ ^239^ ^]^ Webb et al. demonstrated a method to use microfluidics to translate lab‐scale processing to GMP‐grade preparation of protein‐loaded liposomes.^[^ ^240^ ^]^ Hood and co‐workers designed a lab‐on‐a‐chip device based on microfluidics technology to directly prepare drug‐loaded liposomes (Figure 14C).^[^ ^241^ ^]^ In fact, the COVID vaccines are also believed to be manufactured through parallelization of microfluidics systems.^[^ ^242^, ^243^ ^]^

### 4.2 Laser‐Assisted Methods

Among various techniques to prepare micro‐ and nanomaterials, laser‐assisted methods occupy a unique sector. The focused laser beam possesses the capability to deliver sufficient energy, thereby initiating chemical reactions and causing physical changes such as melting and vaporization. Consequently, laser technology can be employed either as a standalone method or in conjunction with other manufacturing techniques, such as laser pyrolysis, to fabricate micro‐ and nanomaterials. From a broader perspective, laser‐assisted preparation of micro‐ and nanostructures, commonly referred to as photolithography, has found wide applications in modern electronics manufacturing and microelectromechanical systems (MEMS).^[^ ^244^, ^245^ ^]^ Although a myriad of methods has been developed, two fundamental attributes of lasers are extensively utilized in these processes.^[^ ^246^, ^247^ ^]^

First, laser energy is harnessed to induce desired effects. One intuitive example is the laser ablation method, which has been comprehensively reviewed in previous works.^[^ ^248^ ^]^ Laser ablation can be performed either in a vacuum chamber, known as pulsed laser ablation (PLA), or in liquid media, as in laser synthesis and processing of colloid in liquids (LSPC). In these processes, the energy delivered by the laser is directly targeted at the material. Laser ablation was primarily employed for processing inorganic materials, such as metals, metal oxides, semiconductive materials, and carbon‐based materials.^[^ ^249^ ^]^ However, heat‐sensitive materials are less compatible due to the high energy associated with laser irradiation.

The second attribute is the high resolution provided by the focused laser beam. A salient application in this category is called direct laser writing.^[^ ^250^ ^]^ It has been widely used in the deposition of metal from pyrolytic or photolytic reactions from their precursors.^[^ ^250^ ^]^ When combined with photoinitiators in polymer resins, laser irradiation can directly induce the polymerization process and fabricate fine structures at sub‐100 nm resolution from polymeric materials.^[^ ^251^, ^252^ ^]^ Other than polymeric materials, carbon‐based materials with micro‐ and nanostructures can also be prepared with laser direct writing.^[^ ^253^ ^]^ Unlike other laser‐based methods that produce particles or fibers as the product, these direct‐writing methods fabricate micro‐ and nanostructures for the applications of flexible electronics, sensors, and microfluidic devices for biomedical applications.

### 4.3 Biological Synthesis (Biosynthesis and Nanobiotechnology)

The recent development of biotechnology, as a novel material preparation method, has attracted interdisciplinary research in multiple areas such as food, energy, and pharmaceutics.^[^ ^254^, ^255^ ^]^ The research on micro‐ and nanomanufacturing received the impact as well. Biotechnology utilizes biological resources including plants, yeasts, and bacteria to produce various types of materials.^[^ ^254^ ^]^ And the biological synthesis of micro‐ and nanomaterials leverages equipment and methods developed in biotechnology. In a few previous reviews, this route of synthesis is also referred to as “biosynthesis,” “green” synthesis, and sometimes “nanobiotechnology”.^[^ ^256^, ^257^, ^258^ ^]^ The preparation of metallic and metal oxide nanoparticles was a major focus in biological synthesis in previous studies.^[^ ^258^ ^]^ Lengke et al. demonstrated the synthesis of silver nanoparticles by *Plectonema boryanum* with silver nitrate as the silver source.^[^ ^259^ ^]^ These nanoparticles are below 40 nm in diameter and their morphology can be modulated by the incubation temperature.^[^ ^259^ ^]^ Metal oxides like iron oxide have been synthesized by *Actinobacter* spp. with potassium ferricyanide and ferrocyanide as the precursor.^[^ ^260^ ^]^ Moreover, the synthesis of CdS quantum crystallites by yeasts and bacteria was reported.^[^ ^261^, ^262^ ^]^ Due to the complexity in biological systems, biological synthesis of micro‐ and nanomaterials is associated with a series of potential reaction mechanisms (e.g., bioreduction from NADH (nicotinamide adenine dinucleotide)).^[^ ^263^ ^]^ Singh et al. reviewed the biological synthesis of metal and metal oxides and discussed key requirements of green synthesis, which fit well with the biological synthesis route.^[^ ^256^ ^]^ Notably, the biosynthesis of natural polymers like polysaccharides, polyamides, and polyesters has been investigated in the past as well.^[^ ^264^ ^]^ These natural polymeric materials, even though they cannot be readily used as micro‐ and nanomaterials, are able to be extracted, processed, and then used as bulk preformed polymers in other micro‐ and nanomanufacturing methods.

## 5 Outlook of Micro‐ and Nanomanufacturing

**Figure 15:**
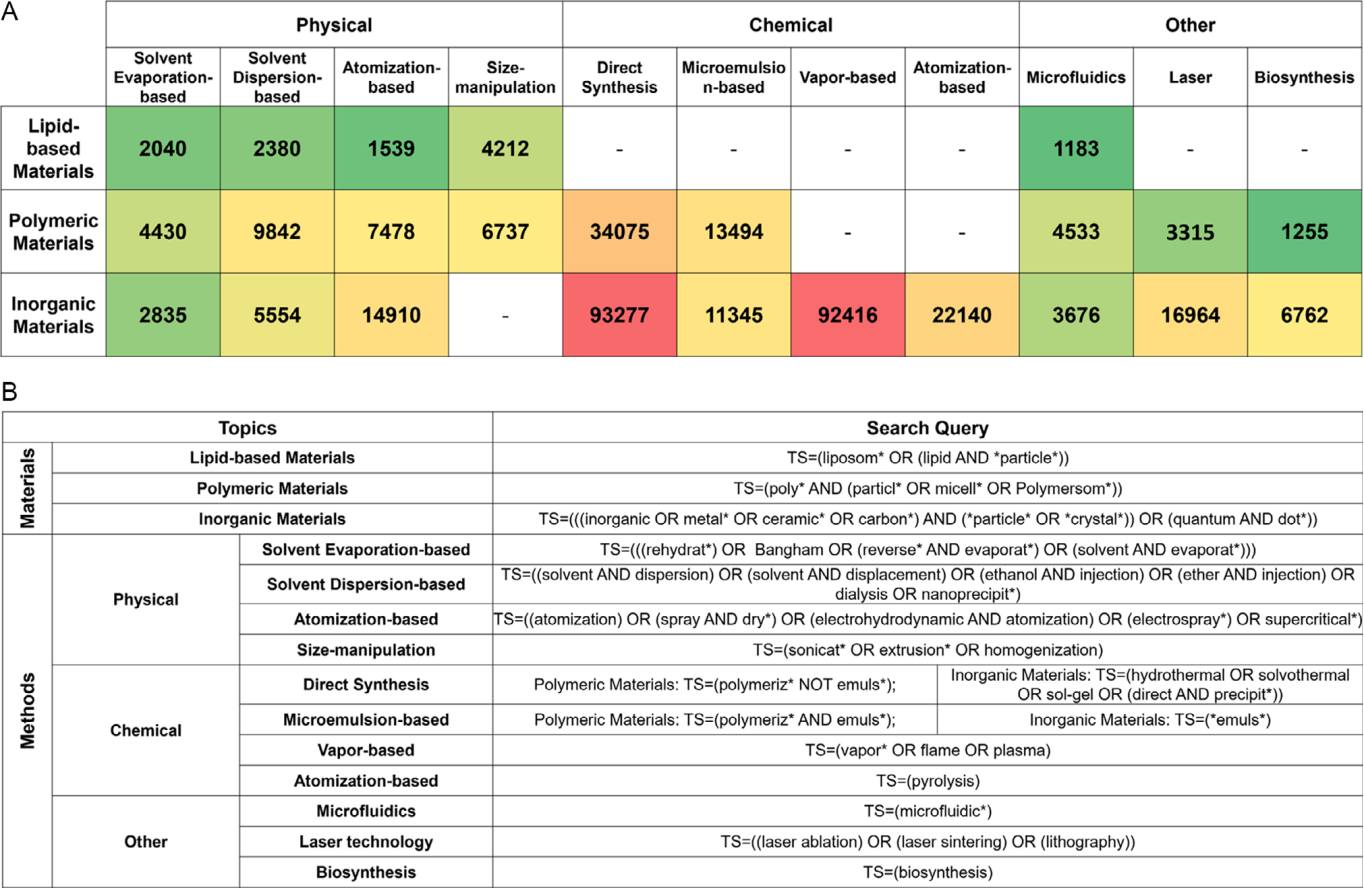
A) A heatmap showing numbers of publications with respect to materials and processing methods. B) Search queries used for obtaining the number of publications on Web of Science. Data retrieved from Web of Science on 2023/03/06.

In the last few decades, urgent demands of precision medicine and advanced diagnostics catalyzed the research in micro‐ and nanomaterials for healthcare applications. To satisfy the need for translating benchtop research to bedside clinical applications, significant efforts have been paid to improve the existing forming methods and to explore novel preparation methods.^[^ ^118^ ^]^ Numerous reviews have covered the preparation methods of micro‐ and nanomaterials in specific domains. In this review, instead of excavating vertically within a specific subdomain of materials, we present a new horizontal viewpoint that uncovers the close relationship between the manufacturing of different micro‐ and nanomaterials. A heatmap containing the total number of publications in each specific domain is presented in **Figure** **15**. As reflected by the figure, the research into processing lipid‐based materials was spread across all physical methods. An emphasis was put on the size manipulation of the prepared liposomes and LNPs. Such observation is also reflected by the fact that the majority of liposomes require further treatment to size‐down and also homogenize before put into applications. In terms of polymeric materials, the focus of research encompasses a wide range of methods from physical to chemical synthesis. Polymeric materials attracted tremendous research interest in exploring their versatility through obtaining different compositions and configurations of polymers with polymerization techniques. Regarding inorganic materials, the research into chemical methods for the preparation outnumbers the physical ones. The chemical and physical stability of inorganic materials than other organic materials could contribute to this difference. In addition, ongoing research efforts are focused on exploring the potential of various advanced technologies, such as microfluidics, laser technologies, and biosynthesis, for the processing of micro‐ and nanomaterials. Microfluidics as a versatile platform technology won favor across different disciplines. In contrast, laser‐assisted synthesis has received an increasing amount of attention in the preparation of inorganic micro‐ and nanomaterials and structures. In the context of commercialized nanomedicines, the selection of appropriate manufacturing methods entails a comprehensive decision‐making process that takes into account several factors, including the characteristics of the encapsulated API, the chosen delivery platform, and the route of administration. To give some examples, DepoCyt, DepoDur, and Exparel share the same delivery technology called DepoFoam, which is composed of micrometer‐sized multivesicular liposomes (Table 1).^[^ ^265^ ^]^ The manufacturing of these multivesicular liposomes, as opposed to smaller unilamellar liposomes, only requires emulsification and does not require further sizing down.^[^ ^265^ ^]^ However, a large particle size is not a favored characteristic when used for intravenous infusion administration.^[^ ^7^ ^]^ Examining APIs loaded into the nanomedicine can help reveal the answer. DepoCyt contains cytarabine and has its indication for lymphomatous or leukemic meningitis, where the lymphoma/leukemia affects the central nervous system. Opioid‐containing DeepDur and bupivacaine‐containing Exparel are both for postsurgery pain management. And all of these indications are associated with the local delivery of APIs instead of delivering through blood circulation. It turned out that DepoCyt, DepoDur, and Exparel are clinically approved for intrathecal, epidural, and subcutaneous administration respectively.^[^ ^266^ ^]^ Indeed, the interplay between the design, manufacture, and application complicates the development of nanomedicine and the actual choice of micro‐ and nanomanufacturing methods. The readers are referred to the review by Anselmo and Mitragotri for the clinical indications of the corresponding nanomedicines.^[^ ^13^ ^]^

### 5.1 Progresses of Nanomedicine Manufacturing in Recent Decades

One topic that cannot be circumvented in the discussion about manufacturing micro‐ and nanomaterials is the difficulty in scaling‐up. It was believed to be one of the major obstacles lying on the path toward the translation of micro‐ and nanomaterials from bench to bedside. Indeed, a major focus of research was localized on exploring the fabrication of materials with novel structures, morphologies, and functions. And the practicality of scaling‐up was sometimes neglected and was not a primary consideration. However, one cannot deny the solid progress in the realm of micro‐ and nanomanufacturing. Sound evidence can be found in the comparison between the stories of doxorubicin hydrochloride liposome and the mRNA COVID vaccine. In 2011, the manufacture of anticancer drug Caelyx, which was a pegylated liposomal injection containing doxorubicin hydrochloride, was ceased due to quality issues identified at the site of production.^[^ ^267^ ^]^ This directly led to a shortage in supply of doxorubicin hydrochloride liposome products until 2013 when a generic drug produced by Sun Pharma was approved by the FDA.^[^ ^268^, ^269^ ^]^ During this period, patients around the world were suffering from the long waiting for any possible source of Caelyx supply. As reviewed by Shah et al., the industrial manufacturing of the evergreen drug delivery platform liposome relies on time and energy consuming multistep batch processing. In addition, a follow‐up homogenization step by membrane extrusion further drives up the cost. The high costs associated with micro‐ and nanomanufacturing challenges pharmaceutical companies and hinders the production of the drug.^[^ ^18^ ^]^ Another story is about the different but close lipid‐based formulation in the COVID‐19 vaccine by Pfizer and Moderna. The urgent demand of these mRNA LNP vaccines during the onset of pandemic. Similarly, the bottleneck of vaccine production was the procedure to mix lipid and mRNAs to formulate LNPs.^[^ ^270^ ^]^ There is no official exposure from Pfizer or Moderna about the manufacturing of LNPs. However, it is widely believed that microfluidics was leveraged, and impingement jet mixing might be the key component of LNP manufacturing.^[^ ^242^, ^270^ ^]^ Impingement jet mixing is a continuous processing technique based on the nanoprecipitation mechanism.^[^ ^271^ ^]^ In fact, the rising of such methods in industrial process can be cross‐confirmed by the fact that the solvent dispersion method (nanoprecipitation) was widely tested and documented in the patents of commercialized lipid‐based nanomedicines dated back to 2018 (Table 1). From batch‐based processes to precisely controlled microfluidic systems for continuous manufacturing, these two examples highlighted the development in the manufacturing process of lipid‐based materials.^[^ ^18^, ^118^, ^272^ ^]^

Another encouraging observation found from the horizontal comparison is in polymeric materials. Unlike the popular and established research and manufacturing pipeline of lipid‐based materials, polymeric particles are much less spoken of in terms of commercialized products. A heated research topic as it is, only a handful of polymer‐based nanomedicines were granted FDA approvals.^[^ ^13^ ^]^ This is partly due to a later onset of research for its application in healthcare, especially as drug delivery platforms, when compared with lipid‐based systems. The safety also cast shadows on the research translatability of polymeric particles. However, it is believed that polymeric nanomaterials, with their innate versatility, will eventually surmount the roadblocks lying in‐between the laboratory development and clinical transition. In fact, a growing number of polymeric nanoparticles including polymeric micelles are currently under clinical evaluation.^[^ ^13^, ^273^ ^]^ In addition, the similarities in the manufacturing processes of various materials made it possible to interchange the accumulated knowledge and even manufacturing facilities between polymeric materials and lipid‐based materials.

This could be especially beneficial for polymeric nanomaterials when they ultimately reach bedside clinical application. As an example, the techniques for expanding production and ensuring quality control that have been acquired through the manufacture of mRNA vaccines with impingement jet mixers can potentially be applied to the large‐scale preparation of polymeric micelles in the future, as the fundamental mechanism of nanoprecipitation/solvent dispersion is similar.^[^ ^274^ ^]^

Such similarities among various processing methods not only revealed the commercialization potential, but also can be inspirational in encouraging interdisciplinary research. The underlying nucleation mechanism is similar in‐between the precipitation preparation of metal oxide particles and the precipitation polymerization of organic monomers. And the word “polymerization” is not exclusively used in polymer chemistry but also an important process in the sol–gel processing. Furthermore, albeit vapor‐based methods seem to be exclusively used by metal oxides, the preparation of carbon‐based materials, including graphene oxide and carbon nanotubes, also rely heavily on these processes.^[^ ^275^ ^]^ These similarities in processing methods again highlighted the interchangeable knowledge across disciplines and their catalyst role in the development of materials preparation.

### 5.2 Sustainability Considerations in Manufacturing

Research in micro‐ and nanomanufacturing has made substantial progress to make the manufacturing process more environmental‐friendly and sustainable.^[^ ^1^, ^276^, ^277^ ^]^ The sustainability of manufacturing processes has now become an important consideration on top of both the practicality and energy consumption during the manufacturing process. Sustainable manufacturing of materials could be improved in multiple aspects, as suggested by Anastas and Zimmerman in the “12 principles of engineering”.^[^ ^278^ ^]^ From the perspective of raw materials, lipid‐based materials typically have a biological origin—most of lipids and cholesterols, which are key components of liposomes and LNPs, are extracted from plant or animal sources (e.g., soybeans and egg yolk).^[^ ^279^ ^]^ Polymeric materials cover a wide range of materials with natural or synthetic origin. Also, a significant amount of recent research effort was spent on discovering biodegradable and recyclable polymers.^[^ ^280^ ^]^Similarly, efforts were made to identify less harmful and energy‐demanding reagents for inorganic micro‐ and nanomaterials. On the other hand, innovations that contribute to the sustainability in manufacturing are not limited to the choice of raw materials but also the processing method. For instance, in thin film rehydration method for liposome preparation, lipids must be dissolved in a solvent and then brought down to a film in the flask. And a “go‐to” solvent in this scenario is chloroform. It is because ingredients of liposome have good solubility profiles in these halogenated solvents like chloroform, whereas the inherent toxicity and carcinogen nature cast shadow on the use of chloroform. Being a class 2 solvent, regulatory bodies pose strict limitations to the residue of chloroform in biomedical products, which require further steps in manufacturing to eliminate the solvent residue.^[^ ^119^ ^]^ The solvent injection method, when compared with solvent evaporation, offered a more favorable and sustainable way for liposome production, making it a new “fashion” in the up‐scaling of recent lipid‐based materials (Table 1). Likewise, developments in processing methods for inorganic materials showed positive impact on sustainability: the solvothermal method significantly reduced the demanding high temperature to sinter ceramic micro‐ and nanomaterials.^[^ ^189^ ^]^ Exemplified by these heartening improvements in manufacturing, it is thus argued that innovations in manufacturing carry critical responsibilities to make the mass production of micro‐ and nanomaterials possible as well as sustainable.

## 6 Conclusions

In recent decades, impressive advancements have been made in the field of micro and nanomaterials for healthcare applications. Through an examination of material preparation methods, this review offers a unique perspective for comparing these methods across various types of micro‐ and nanomaterials. It was observed that correlated forming mechanisms and interchangeable techniques are present across different micro‐ and nanomaterials. Furthermore, for the first time, a table was compiled featuring processing techniques that have been included in the patents of commercially available nanomedicine products. These fabrication techniques in patents reflected the solid development of micro‐ and nanomanufacturing and highlighted the transition from laborious batch‐based methods to efficient continuous production. Finally, based on the similarities in manufacturing processes, it is reasonable to anticipate that the scaling‐up production of other types of nanomedicines, such as polymeric micro‐ and nanoparticles, will benefit from the established experiences of lipid‐based materials, accelerating the bench‐to‐bedside translation.

## Conflict of Interest

The authors declare no conflict of interest.
